# Atomic Layer Deposition of Large-Area Polycrystalline Transition Metal Dichalcogenides from 100 °C through Control of Plasma Chemistry

**DOI:** 10.1021/acs.chemmater.2c01154

**Published:** 2022-08-05

**Authors:** Miika Mattinen, Farzan Gity, Emma Coleman, Joris F. A. Vonk, Marcel A. Verheijen, Ray Duffy, Wilhelmus M. M. Kessels, Ageeth A. Bol

**Affiliations:** † Department of Applied Physics, Eindhoven University of Technology, P.O. Box 513, 5600 MB Eindhoven, The Netherlands; ‡ Tyndall National Institute, University College Cork, Lee Maltings, Dyke Parade, T12 R5CP Cork, Ireland; § Eurofins Materials Science Netherlands, High Tech Campus 11, 5656 AE Eindhoven, The Netherlands; ∥ Department of Chemistry, University of Michigan, 930 N. University Avenue, Ann Arbor, Michigan 48109-1055, United States

## Abstract

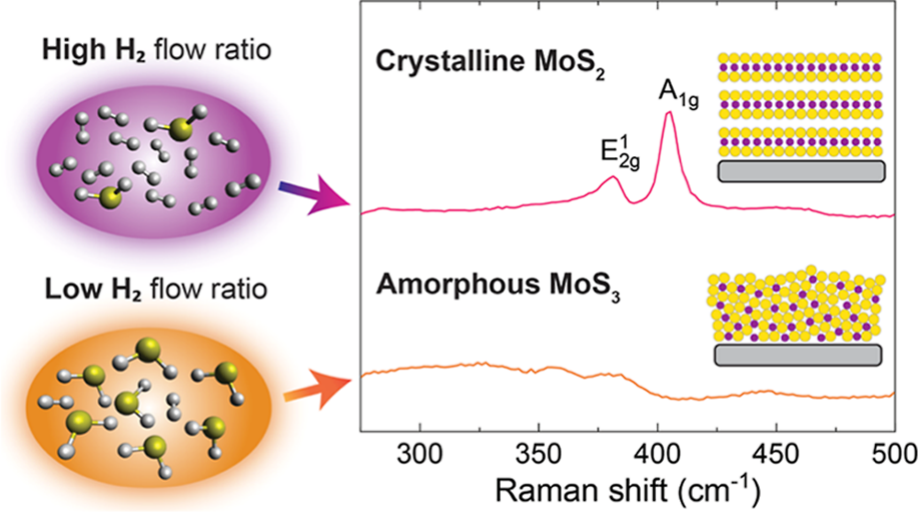

Two-dimensional transition metal dichalcogenides, such as MoS_2_, are intensely studied for applications in electronics. However, the difficulty of depositing large-area films of sufficient quality under application-relevant conditions remains a major challenge. Herein, we demonstrate deposition of polycrystalline, wafer-scale MoS_2_, TiS_2_, and WS_2_ films of controlled thickness at record-low temperatures down to 100 °C using plasma-enhanced atomic layer deposition. We show that preventing excess sulfur incorporation from H_2_S-based plasma is the key to deposition of crystalline films, which can be achieved by adding H_2_ to the plasma feed gas. Film composition, crystallinity, growth, morphology, and electrical properties of MoS_*x*_ films prepared within a broad range of deposition conditions have been systematically characterized. Film characteristics are correlated with results of field-effect transistors based on MoS_2_ films deposited at 100 °C. The capability to deposit MoS_2_ on poly(ethylene terephthalate) substrates showcases the potential of our process for flexible devices. Furthermore, the composition control achieved by tailoring plasma chemistry is relevant for all low-temperature plasma-enhanced deposition processes of metal chalcogenides.

## Introduction

Two-dimensional (2D) transition metal dichalcogenides (TMDCs) boast of unique electronic, optical, and mechanical properties as a result of their layered crystal structure.^1−3^ These properties combined with their stability as individual monolayers have attracted a great deal of interest for applications including electronics,^1,3,4^ catalysis,^3,5^ energy storage,^3,6^ and medicine.^3,7^ The diverse TMDC family contains more than 30 layered materials with composition MX_2_ (M = transition metal or Sn; X = S, Se, Te), which range from semiconductors to (semi)metals and even superconductors.^1,2,8,9^ Semiconducting TMDCs, such as MoS_2_ and WS_2_, have drawn particular attention in search for alternatives to silicon for future nanoelectronics. Many TMDC-based (opto)electronic devices with excellent performance have been demonstrated, including field-effect transistors (FETs),^10−12^ photodetectors,^13^ and new device concepts such as tunneling FETs,^14^ memristors,^15,16^ and memtransistors.^17^

In many cases, the available fabrication methods are unable to match the requirements of potential applications. The initial studies on TMDCs and even many investigations today rely on ultrathin flakes mechanically exfoliated from bulk crystals, a method that is inherently unscalable.^3,17,18^ To bring TMDCs closer to applications, high-temperature chemical vapor deposition (CVD) processes producing high-quality material from oxide powder precursors have been developed.^9,19−22^ Many of these processes struggle with uniformity on large areas and thickness control, although some deposition chemistry and engineering solutions to these issues have been presented.^23−27^ What remains largely unsolved, however, is the high temperature required by the CVD processes, typically 600–800 °C for MoS_2_.

Integrating TMDCs into back-end-of-line semiconductor processing, which has a maximum allowed processing temperature of 400–500 °C,^28^ is widely explored in order to add new functionalities such as memory and photodetectors onto Si-based chips.^29−31^ To this end, metal–organic and halide CVD^32−34^ and atomic layer deposition (ALD—a surface-controlled variant of CVD)^35−38^ processes depositing polycrystalline MoS_2_ films at 200–500 °C have been developed. TMDCs are also promising materials for the emerging fields of flexible electronics and displays, where they may be used as thin-film transistors and sensors for gases, light, biomolecules, and pressure, for example.^39−41^ The highest temperature that typical polymer-based flexible substrates withstand range from 100 to 400 °C.^41−43^ For one of the most common and cost-effective plastics, poly(ethylene terephthalate) (PET), temperature should not exceed 100–150 °C to avoid its deformation.^42^ Although a few ALD processes for TMDCs operating at 150 °C or below are known,^36,44−46^ these processes deposit amorphous films, which typically have insufficient conductivity and charge carrier mobility for use in electronics. Thus, for plastic-based flexible electronics, only transfer of films deposited on another substrate^47^ and assembly (e.g., printing or spin coating) from a dispersion of flakes^48^ are currently available. Direct deposition processes are more desirable for process simplicity and cost, film quality and uniformity, and coverage on 3D structures.

Plasma-enhanced atomic layer deposition (PEALD) is capable of producing high-quality films at low temperatures enabled by highly reactive radicals as well as low-energy ions that can provide additional energy to the surface without substantially heating the substrate.^49^ Similar to thermal ALD, PEALD relies on self-limiting surface reactions and therefore offers excellent film uniformity, accurate thickness control, high reproducibility, and facile scalability.^50,51^ For semiconducting MoS_2_ and WS_2_, PEALD processes have been developed in our group that perform well at moderately low temperatures (≥300 °C).^36,52^ However, below 300 °C, the processes produce amorphous, sulfur-rich films that are unsuitable for electronic applications due to their high resistivity. Highly conductive TiS_2_ films, on the other hand, have been deposited down to 150 °C by PEALD.^53^

In this work, we demonstrate PEALD of polycrystalline thin films of MoS_2_, TiS_2_, and WS_2_ with controlled thickness at temperatures down to 100 °C. To our knowledge, this is the lowest temperature reported for MoS_2_ deposited by a chemical vapor-phase method. We have identified two key factors enabling the low-temperature deposition. The first is the use of remote H_2_S-based plasma to produce highly reactive species including radicals and low-energy ions. The second factor is providing sufficient H_2_ together with the sulfur source H_2_S to limit S incorporation, as excess S inhibits crystallization. Besides stoichiometry and crystallinity, hydrogen is observed to affect film growth, morphology, and electrical properties as examined in detail for MoS_*x*_. In particular, we investigate the properties of MoS_2_ films deposited at the lowest temperatures that are compatible with deposition on plastic substrates used in flexible electronics. In addition, we discuss key experimental process parameters, which hold not only for MoS_2_ but also for other chalcogenides prepared by plasma-enhanced processes.

## Results and Discussion

### Concept and Effect of H_2_ on Crystallinity

**Figure 1:**
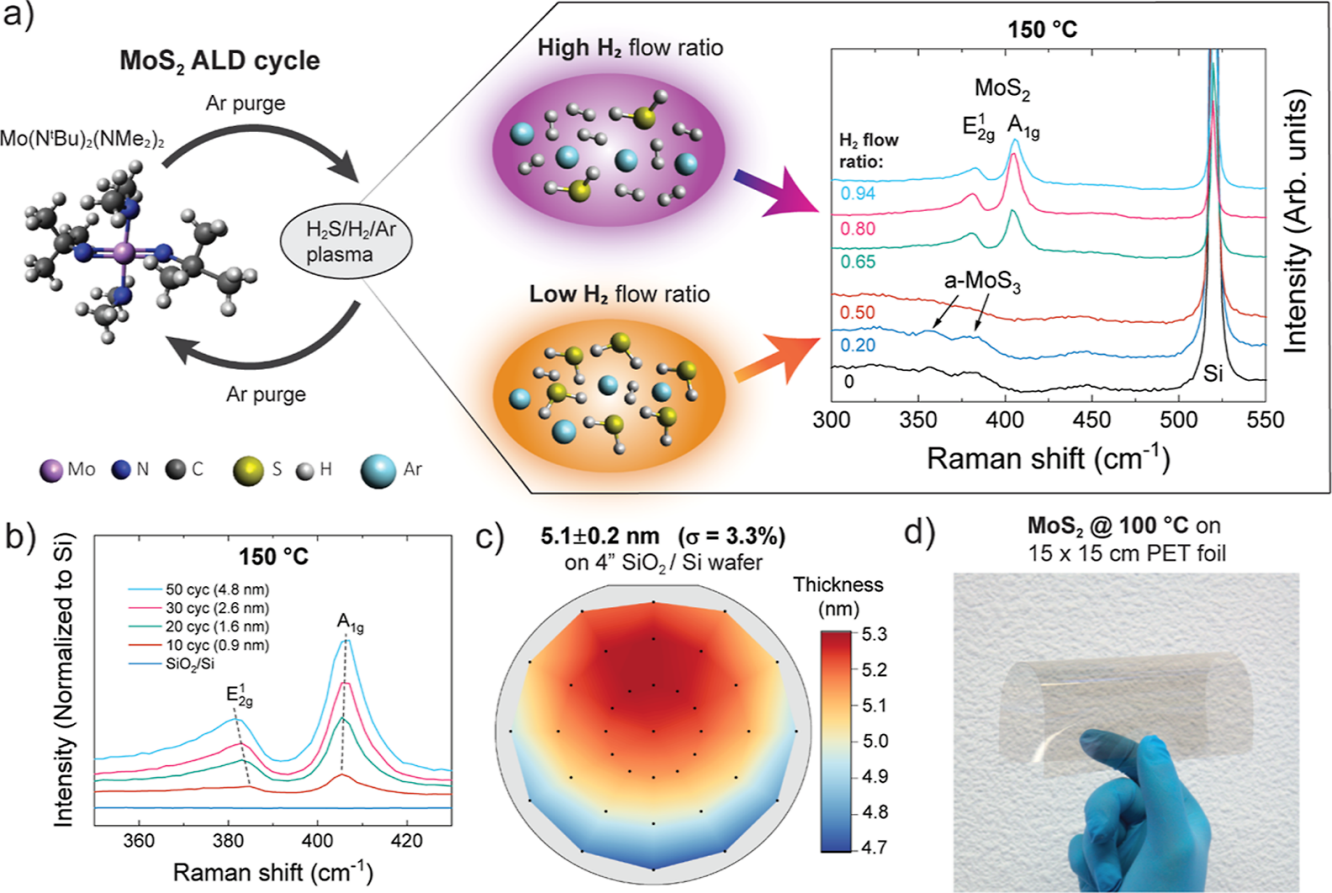
Illustration of the PEALD process and its ability to deposit crystalline, wafer-scale MoS_2_ films of controlled thicknesses at low temperatures. (a) Schematic of the process and Raman spectra showing the effect of the H_2_ flow ratio on crystallinity of MoS_*x*_ films (150 °C, 140 ALD cycles). (b) Raman spectra and ex situ SE thicknesses illustrating crystallinity and accurate thickness control. (c) SE thickness map over 4″ wafer (dots represent data points, film grown at 150 °C, H_2_ flow ratio of 0.80, 50 ALD cycles). (d) Photograph of an approximately 5 nm thick MoS_2_ film deposited on a transparent PET substrate (15 × 15 cm) at 100 °C using a H_2_ flow ratio of 0.80 and 50 ALD cycles.

Our PEALD process illustrated in Figure 1a begins by pulsing a metal–organic molybdenum precursor Mo(N^*t*^Bu)_2_(NMe_2_)_2_ vapor into the ALD reactor, which then reacts with the substrate surface until all reactive surface sites are consumed. Next, excess precursor and byproducts are removed in a purge step. Then, the substrate is exposed to a mixed H_2_S/H_2_/Ar remote plasma that serves to remove the ligands of the adsorbed Mo precursor as well as to supply S into the films. After a second purge step, an ALD cycle is complete. The cycle can be repeated as many times as desired to reach the target thickness. Saturation curves confirming the self-limiting nature of our ALD process are presented in Supporting Information (Figure S1).

The plasma feed gas (H_2_S/H_2_/Ar) composition together with the deposition temperature determine whether amorphous S-rich (a-MoS_2+*x*_) or polycrystalline MoS_2_ (c-MoS_2_) films are deposited. The fraction of H_2_ in the plasma feed gas, that is *H*_*2*_*flow ratio*, is defined by the gas flow rates as shown in eq 1. The absolute flow rates and other experimental parameters are described in the Experimental Section.
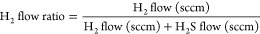
1

At a low temperature of 150 °C, a-MoS_2+*x*_ is deposited when a low H_2_ flow ratio from 0 to 0.50 is used, which results in weak and broad Raman modes similar to those reported for amorphous MoS_3_ (a-MoS_3_, Figure 1a).^54^ In contrast, polycrystalline MoS_2_ can be deposited using a higher H_2_ flow ratio of at least 0.65, as is evident from the emergence of the E_2g_^1^ and A_1g_ Raman modes of MoS_2_. The intensity of the Raman peaks further increases going from a H_2_ flow ratio of 0.65–0.80, which suggests improved crystallinity. The crystallinity of the films deposited at H_2_ flow ratios of at least 0.65 was confirmed by X-ray diffraction (Figure S2 in Supporting Information).

With an optimized H_2_ flow ratio of 0.80, we are able to deposit crystalline MoS_2_ films with submonolayer-level thickness control at 150 °C (Figure 1b). Characteristic MoS_2_ Raman modes can be identified even for an approximately 1 nm film deposited using 10 ALD cycles. When the number of ALD cycles and thus the film thickness is increased, the E_2g_^1^ and A_1g_ peaks shift to lower and higher wavenumbers, respectively, confirming accurate thickness control.^55,56^ By increasing the number of ALD cycles, the film thickness can be easily controlled from approximately one monolayer to tens of nanometers. Throughout this article, the effect of the H_2_ flow ratio on growth and various properties of MoS_*x*_ films is mainly discussed using 10–20 nm thick films prepared using 100–140 ALD cycles.

Our process deposits films with excellent wafer-scale uniformity, as illustrated in Figure 1c with a thickness standard deviation (σ) of 3.3% over a 4″ wafer. Atomic force microscopy (AFM) showed that approximately 1–5 nm thick MoS_2_ films had low roughnesses and appeared continuous without any visible holes (Figures S3 and S4 in Supporting Information). Using the lowest deposition temperature of 100 °C and an optimized H_2_ flow ratio of 0.80, MoS_2_ films can be deposited on low-cost plastic substrates applicable for flexible electronics such as PET as shown in Figure 1d. Approximately 5 nm thick MoS_2_ films deposited on PET were highly transparent and had a relatively low resistivity of 0.3 Ω cm, which was unchanged after repeated bending by hand. The electrical properties of the films will be discussed in detail in a dedicated section below.

**Figure 2:**
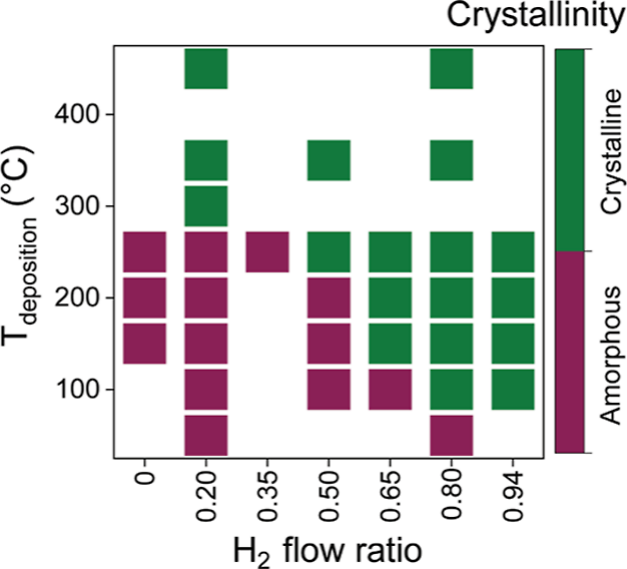
Effect of H_2_ flow ratio and deposition temperature on film crystallinity as analyzed by Raman spectroscopy. The films were deposited using 100–140 ALD cycles.

The effect of both the H_2_ flow ratio and deposition temperature on film crystallinity is shown in Figure 2 (for the Raman spectra used to construct the heat map see Figure S5 in Supporting Information). It is apparent that the minimum H_2_ flow ratio required to deposit crystalline films increases with decreasing deposition temperature. At ≥300 °C, even a low H_2_ flow ratio of 0.20 deposits c-MoS_2_, whereas at 100 °C a H_2_ flow ratio of 0.80 is required to deposit c-MoS_2_. In addition to a minimum H_2_ flow ratio for each temperature, an upper bound for the H_2_ flow ratio was also identified. Although not obvious from the Raman spectra, X-ray photoelectron spectroscopy (XPS) indicated that a H_2_ flow ratio of 0.94 was too high as will be discussed in the following sections.

**Figure 3:**
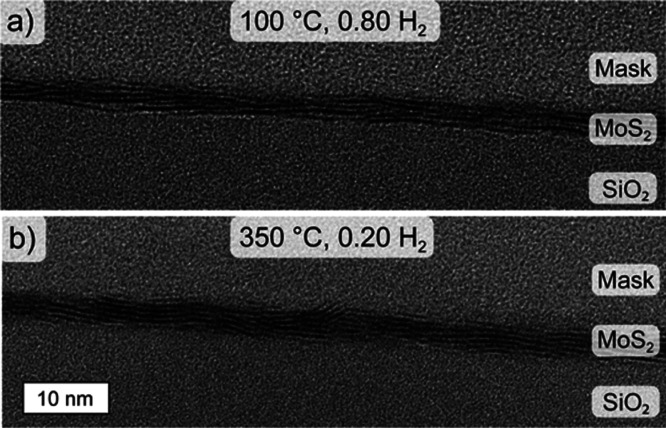
Film microstructure. Cross-sectional TEM images of films grown at (a) 100 °C using a H_2_ flow ratio of 0.80 and 50 ALD cycles (lowest temperature for c-MoS_2_) and (b) 350 °C using a H_2_ flow ratio of 0.20 and 60 ALD cycles (reference condition following Sharma et al.^36^).

Cross-sectional transmission electron microscopy (TEM) was used to examine the crystallinity and microstructure of the films. For this purpose, we deposited a sub-5 nm c-MoS_2_ film at the lowest temperature of 100 °C using a H_2_ flow ratio of 0.80 and 50 ALD cycles. The film appears fully crystallized and is composed of 4–5 monolayer thick and approximately 5–10 nm wide nanocrystallites (Figure 3a). The film surface is relatively smooth with a root-mean-square roughness of 0.75 nm measured by AFM. Furthermore, this 100 °C film was compared to a reference film deposited at a higher temperature of 350 °C and a lower H_2_ flow ratio of 0.20 (Figure 3b). Remarkably, the microstructure and crystallinity of this 5–6 monolayer film deposited at 350 °C is rather similar to the 100 °C film.

### Film Composition

**Figure 4:**
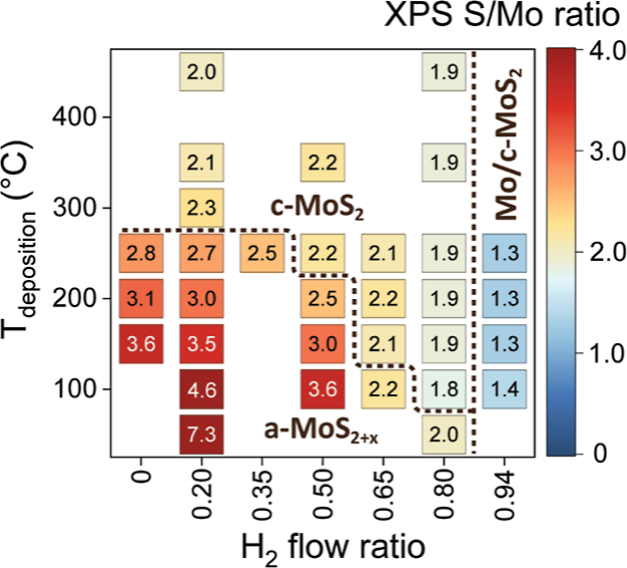
Effect of the H_2_ flow ratio on film stoichiometry and correlation of stoichiometry and crystallinity as shown by a heat map of S/Mo atomic ratio analyzed by XPS (the three regions identified are highlighted). The films were deposited using 100–140 ALD cycles.

A striking correlation between the S/Mo atomic ratio and crystallinity was found as shown in Figure 4. The composition of the majority of the crystalline films was close to MoS_2_, ranging from MoS_1.8_ to MoS_2.3_ by XPS. Thus, the correct stoichiometry and chemical environment (discussed below) achieved by controlling plasma chemistry enable crystallization of MoS_2_ at temperatures as low as 100 °C. In contrast, the amorphous films deposited at ≥150 °C contained excess S (MoS_2.5_ to MoS_3.6_). In the amorphous region, an increase of temperature at a constant H_2_ flow ratio resulted in a decrease of the S/Mo ratio, which is likely due to changes in reaction mechanisms and stability of S^2–^ and S_2_^2–^ species as a function of temperature. Namely, more sulfur-rich compositions and the sulfur species contained within are less stable at higher temperatures (to be discussed below and in Section S14 in Supporting Information). Thus, the control of stoichiometry and crystallinity is a result of interplay of plasma and surface chemistries—the former controlled by the H_2_ flow ratio and the latter by the H_2_ flow ratio, deposition temperature, and the crystallinity of the deposited film.

Two exceptions to the stoichiometry–crystallinity correlation were identified. First, at the lowest temperatures of 50–100 °C, some of the films grown using high H_2_ flow ratios were close to MoS_2_ stoichiometry yet remained amorphous. This can be due to insufficient thermal energy for crystallization as well as the chemical environment of S being different to that of MoS_2_ and more representative of a-MoS_2+*x*_ as discussed below. Second, using the highest evaluated H_2_ flow ratio of 0.94 resulted in a low S/Mo ratio despite Raman spectroscopy showing MoS_2_ peaks. As discussed below, this is due to the films consisting of both c-MoS_2_ and metallic Mo components, suggesting that too high a H_2_ flow ratio can result in insufficient S supply and/or excessive etching of S from MoS_*x*_ films.

**Figure 5:**
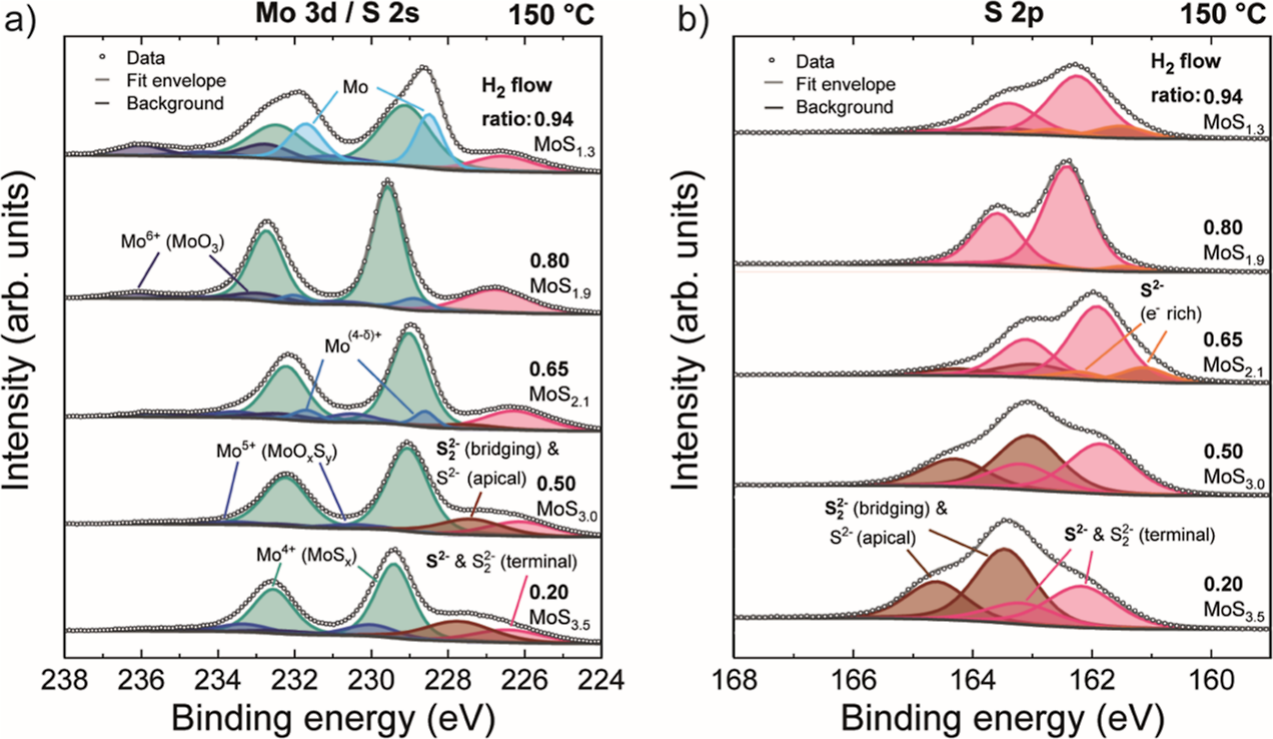
Effect of the H_2_ flow ratio on the chemical environment of Mo and S. XPS spectra of (a) Mo 3d/ S 2s and (b) S 2p core levels of MoS_*x*_ films grown at 150 °C using different H_2_ flow ratios and 140 ALD cycles. S/Mo stoichiometries are also indicated.

The chemical environment of molybdenum and sulfur was analyzed using core level X-ray photoelectron spectra. Films deposited at 150 °C using different H_2_ flow ratios will be discussed as an example. The results for other temperatures can be found in Supporting Information (Table S1). Analysis of the Mo 3d region showed that the majority of Mo was present as Mo^4+^, which is attributed to MoS_*x*_ species (Figure 5a). Two additional doublets at higher binding energies (BEs) with lower intensities were observed in all samples. The highest BE doublet is attributed to Mo^6+^ (MoO_3_) as a result of post-deposition oxidation in air.^57^ The doublet between the Mo^4+^ and Mo^6+^ doublets is assigned to Mo^5+^ (MoO_*y*_S_*x*_), an intermediate oxidation product.^58,59^ For c-MoS_2_ films, an additional low-intensity doublet at a lower BE compared to Mo^4+^ was required to obtain a satisfactory fit. This doublet is denoted as Mo^(4−δ)+^ as it was attributed to more metallic Mo atoms present at the edges of MoS_2_ crystals,^60^ in the metastable 1T′ phase of MoS_2_,^61^ or MoS_2–*x*_ subsulfide.^62^

Using the c-MoS_2_ film grown with a H_2_ flow ratio of 0.80 as an example, the Mo 3d_5/2_ BEs were 228.9 eV (Mo^(4−δ)+^), 229.6 eV (Mo^4+^), 231.1 eV (Mo^5+^), and 232.9 eV (Mo^6+^). The observed BEs are in good agreement with average literature values for MoS_2_ (229.3 ± 0.5 eV) and MoO_3_ (232.7 ± 0.3 eV).^63^ Slight variation in measured BEs depending on the deposition conditions was observed as discussed in Supporting Information (Section S5). Furthermore, for the highest H_2_ flow ratio of 0.94, an intense doublet at a BE below that of Mo^4+^ was identified, which was assigned to metallic Mo.

In addition to Mo 3d, two S 2s singlets that partially overlap with Mo 3d were fit to the data. A more detailed view of the S species can be obtained from the S 2p region. For the crystalline MoS_2_ film deposited at a H_2_ flow ratio of 0.80, the S 2p spectrum could be fit rather well using a single doublet attributed to S^2–^ in MoS_2_ (Figure 5b, S 2p_3/2_ BE = 162.4 eV). Inclusion of a low-intensity doublet at approximately 0.9 eV lower BE further improved the fit, which we attribute to electron-rich S^2–^ species that may be present on metallic edges of MoS_2_ crystals, in 1T′ MoS_2_ or MoS_2–*x*_ subsulfide (see Mo 3d discussion above).^61,62^

An additional doublet at a higher BE compared to S^2–^ was especially pronounced for lower H_2_ flow ratios producing amorphous films. For example, in an a-MoS_3.0_ film grown at a H_2_ flow ratio of 0.50, the S 2p_3/2_ BEs of the two species were 161.9 and 163.1 eV, and both doublets had approximately equal areas (the electron rich S^2–^ species were not observed for this and other amorphous samples). The two doublets are in accordance with the literature on amorphous MoS_3_, a material that has been identified to contain sulfur in at least four different chemical environments.^64,65^ The lower BE doublet represents both MoS_2_-like S^2–^ species and a variety of disulfide species denoted as terminal S_2_^2–^. The higher BE doublet consists of bridging S_2_^2–^ and apical S^2–^ species. The heterogeneous nature of sulfur species and their overlapping BEs prevent accurate determination of the S species or even S oxidation states present. However, it was found that the areal ratio of the higher BE doublet to lower BE doublet increased for more sulfur-rich material. Considering that Mo is present as Mo^4+^ in both MoS_2_ and MoS_3_, the more sulfur-rich MoS_2+*x*_ is, the more S_2_^2–^ instead of S^2–^ it must contain to retain charge neutrality. Thus, the main contribution to the observed lower and higher BE doublets must come from S^2–^ and S_2_^2–^ species, respectively.

To confirm the S/Mo stoichiometry analyzed by XPS as well as to gain insights into impurities, Rutherford backscattering spectrometry (RBS) and elastic recoil detection (ERD) measurements were performed on three samples deposited at 150 °C, one amorphous (H_2_ flow ratio of 0.20) and two crystalline films (H_2_ flow ratios of 0.65 and 0.80). The measured S/Mo ratios of the films are in close agreement with XPS (Table 1). In terms of impurities, the nitrogen content resulting from remaining precursor ligands was rather low, 0.5–1.5 at. %. The oxygen content was below 1 at. % in the films grown using H_2_ flow ratios of 0.20 and 0.65. Using a higher H_2_ flow ratio of 0.80, the O content was higher at 5 at. %, which is likely due to surface oxidation combined with rough morphology exposing abundant, easily oxidized edge sites that will be discussed below and is also suggested by the low mass density. The most significant impurity was hydrogen, the concentration of which increased from 6 at. % at the lowest H_2_ flow ratio of 0.20 to 12 and 22 at. % at H_2_ flow ratios of 0.65 and 0.80, respectively. Although the rough morphology and surface oxidation of the last film may somewhat increase the value, the trend of increasing H content in the films with an increasing H_2_ flow ratio is clear. The H impurity will be discussed further in the context of *electrical properties*.

**Table 1:** Effect of the H_2_ Flow Ratio on Film Composition^a^

H_2_ flow ratio	S/Mo ratio (RBS)	S/Mo ratio (XPS)	density (g/cm^3^)	H (at. %)	C (at. %)	N (at. %)	O (at. %)
0.20	3.7 ± 0.1	3.4 ± 0.2	3.2	6.4 ± 0.5	<dl.	0.5 ± 0.2	0.8 ± 0.2
0.65	2.1 ± 0.1	2.1 ± 0.1	4.0	11.7 ± 0.9	<dl.	1.5 ± 0.4	0.5 ± 0.1
0.80	1.8 ± 0.1	1.9 ± 0.1	3.1	22 ± 2	<dl.	1.0 ± 0.3	5 ± 2

a Composition of ∼50 nm thick MoS_*x*_ films deposited at 150 °C using H_2_ flow ratios resulting in amorphous (H_2_ flow ratio 0.20) and crystalline (H_2_ flow ratios of 0.65 and 0.80) films as analyzed by RBS (Mo, S, C, N, O) and ERD (H). Detection limit (dl.) for C is approximately 7 at. % for these films grown on a glassy carbon substrate. S/Mo ratio determined by XPS is shown for comparison. The density was calculated using areal atom densities determined by RBS and thicknesses determined by SE. Bulk density of MoS_2_ is 5.06 g/cm^3^.^66^

### Film Morphology

**Figure 6:**
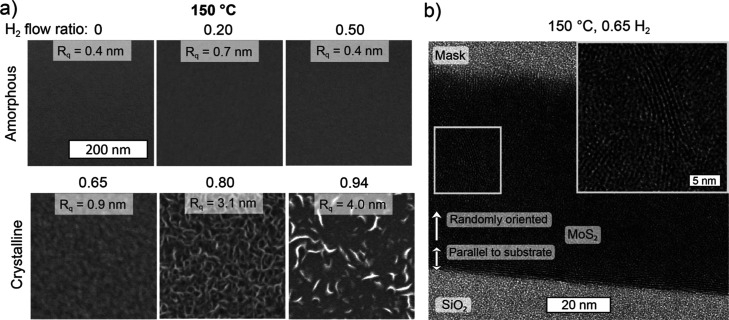
Effect of the H_2_ flow ratio on film morphology and microstructure. (a) SEM images and root-mean-square roughness values (*R*_q_) measured by AFM for films grown at 150 °C with different H_2_ flow ratios (8–20 nm thick films grown using 140 ALD cycles). (b) Cross-sectional TEM image of a ∼50 nm thick MoS_2_ film grown at 150 °C using a H_2_ flow ratio of 0.65 and 500 ALD cycles. The inset shows a magnification of the area marked with a gray square to better visualize the randomly oriented 2D layers. A smoothing filter has been applied to the image—an unfiltered image is presented in Supporting Information (Figure S9).

Film morphology is an important film characteristic that affects film properties. For example, a rough surface can lead to an increase in resistivity due to scattering of charge carriers and affect the ability to integrate films into nanoscale devices. Furthermore, morphology can provide insights into growth mechanisms. For this purpose, relatively thick (8–20 nm) films deposited using 140 ALD cycles are discussed. Thinner films are smoother and show less differences between different conditions. At 150 °C, amorphous films deposited using low H_2_ flow ratios ranging from 0 to 0.50 were very smooth according to scanning electron microscopy (SEM) and AFM (Figure 6a). Increasing the H_2_ flow ratio to 0.65 produced crystalline yet smooth films. In contrast, upon a further increase of the H_2_ flow ratio to 0.80, rough films consisting of out-of-plane oriented MoS_2_ crystallites or “fins” were deposited. At the highest H_2_ flow ratio of 0.94, the areal density of fins decreased, which may be due to the film partially consisting of metallic Mo besides MoS_2_. Similar observations were made for other deposition temperatures keeping in mind that the minimum H_2_ flow ratio required to grow crystalline films, which tend to have a rougher morphology, decreases with increasing deposition temperature. Amorphous MoS_2+*x*_ films grown at all temperatures were smooth (Figure S8 in Supporting Information). Crystalline MoS_2_ films grown just above the crystallization onset were relatively smooth, and increasing the H_2_ flow ratio led to rougher morphology such that the roughest films were observed at a H_2_ flow ratio of 0.80. Thus, a higher H_2_ flow ratio seems to favor fin formation and/or growth.

*Formation* of out-of-plane oriented fins has been commonly observed for (PE)ALD as well as CVD grown TMDC multilayer films (see refs (35) and (67) and references therein). In the case of PEALD MoS_2_ and WS_2_, the fins have been observed to be located at grain boundaries.^36,52,67^ One potential mechanism causing fins to form at the grain boundaries results from two adjacent TMDC crystallites growing laterally until they meet each other, after which the topmost TMDC layer(s) in one or two of the grains involved bend upward forming a fin. Another possibility is that the metal precursor may adsorb onto highly reactive sites in a grain boundary, or another defect site, forming a new grain with presumably a random orientation. Considering the fins have been found to originate almost exclusively at grain boundaries in PEALD TMDCs, a change in grain size and thus grain boundary density affects fin density.^67^

Besides fin formation, fin *growth* also affects the resulting film morphology. In general, fin edges have a larger density of reactive sites compared to basal planes. Therefore, formation of a new MoS_2_ layer on top of a basal plane is slow compared to vertical growth of fins, which explains why the fins grow faster than the basal planes in the vertical direction. This, in turn, results in the often observed transition from a smooth, flat film to rough, fin-dominated film with increasing thickness. In the case of our process, the H_2_ flow ratio can affect the reactivity of fin edges by modifying the coverage of sulfur and/or sulfur-bound hydrogen (S–H) on the edges. Previously, density functional theory (DFT) calculations for WS_2_ have shown that a lower S coverage as well as a higher S–H coverage—both of which may result when increasing the H_2_ flow ratio—makes adsorption of W precursor on the edge more favorable.^52^ A similar effect may also be expected in our process considering that our Mo precursor Mo(N^*t*^Bu)_2_(NMe_2_)_2_ contains identical ligands to the W precursor investigated by DFT, as well as the fact that Mo and W are chemically similar.

As described above, thicker films grown using a high H_2_ flow ratio of 0.80 have a very rough morphology, which suggests that fins can both form and grow easily under these conditions. In contrast, using a slightly lower H_2_ flow ratio of 0.65 at 150 °C results in crystalline films that are smooth even at a thickness of approximately 50 nm. Such a film was investigated by TEM, which revealed that for the first ∼5 nm of the film closest to the substrate, MoS_2_ grains were oriented approximately parallel to the SiO_2_ substrate (Figure 6b). Above that, however, the film consisted of randomly oriented crystallites, which would be expected to form fins. In this case, however, a smooth, dense film was formed. A possible explanation is that under relatively S-rich (i.e., H-poor) plasma conditions the growth of fins is hindered relative to S-poor (H-rich) conditions. Furthermore, our results suggest that the H_2_ flow ratio where this transition occurs depends on temperature such that at a higher deposition temperature a lower H_2_ flow ratio is sufficient to “activate” the edges for precursor adsorption and prominent fin growth. This, in turn, may be due to kinetics of Mo(N^*t*^Bu)_2_(NMe_2_)_2_ adsorption on edges and/or the fact that excess S is more easily present on the surface at lower temperatures. The latter is in line with the observation that a higher H_2_ flow ratio is required at lower temperatures to achieve MoS_2_ stoichiometry and thus crystallinity.

### Film Growth

**Figure 7:**
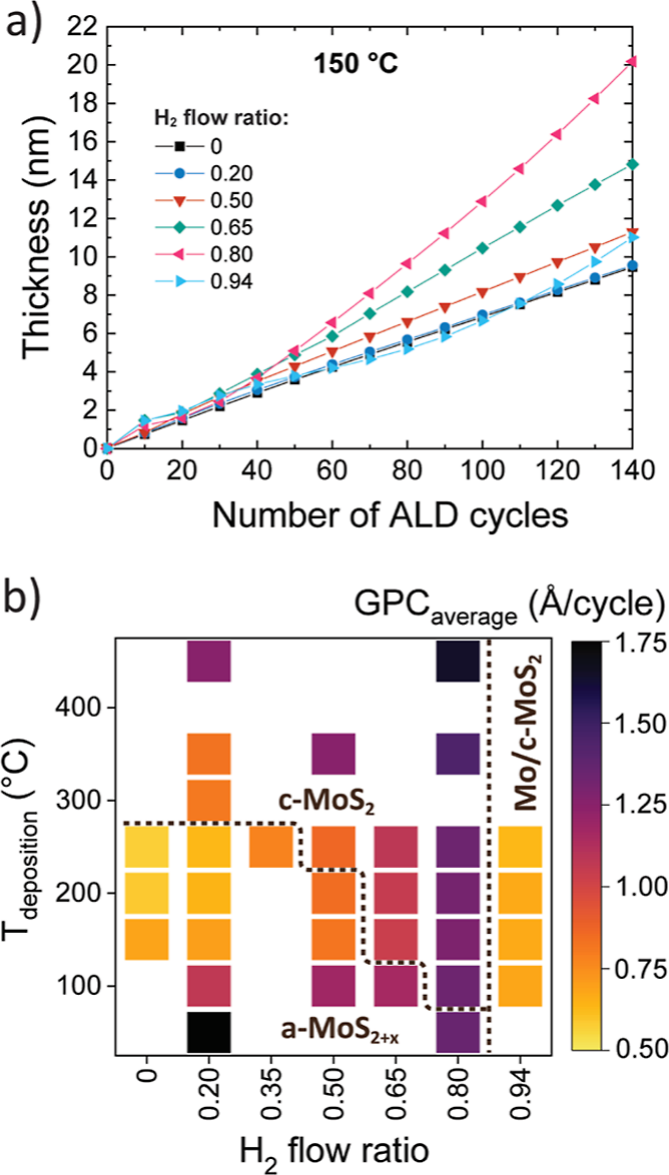
Effect of the H_2_ flow ratio on film growth. (a) Evolution of film thickness measured by in situ SE at 150 °C using different H_2_ flow ratios. (b) Heat map of GPC (averaged over first 100 cycles) as a function of deposition temperature and the H_2_ flow ratio.

In situ SE measurements revealed that the thickness of the amorphous films deposited using H_2_ flow ratios of 0 to 0.50 at 150 °C increased linearly as a function of ALD cycles (Figure 7a shows thickness vs cycles, Figure S10 in Supporting Information shows growth per cycle (GPC) vs cycles). Crystalline films grown just above the crystallization onset (H_2_ flow ratio of 0.65 at 150 °C) also grew rather linearly, although with an increased GPC compared to amorphous films. A further increase of the H_2_ flow ratio to 0.80 resulted in clearly non-linear growth, such that the GPC increased with increasing number of ALD cycles. Finally, an increase of the H_2_ flow ratio to 0.94 led to a decrease in GPC, which may be linked to the film consisting of a mixture of MoS_2_ and Mo phases that likely have different GPCs due to different physical and atom densities, reaction mechanisms, morphologies, and so forth.

Plotting the GPC averaged over the first 100 ALD cycles shows that the GPC trends were similar at all temperatures. Namely, GPC increased when increasing the H_2_ flow ratio from 0 to 0.80 (Figure 7b, in situ SE data shown in Figure S11 in Supporting Information). We attribute the observed trend to two factors.

First, a higher H_2_ flow ratio seems to provide more adsorption sites for the Mo precursor, resulting in more Mo atoms being deposited per cycle. This argument is supported by RBS, which showed that the number of Mo atoms deposited per cycle more than doubled from 0.6 to 1.6 at/nm^2^/cycle when increasing the H_2_ flow ratio from 0.20 to 0.65 at 150 °C while the morphology did not change drastically. This can be understood considering that prior DFT studies for WS_2_ PEALD have shown that metal precursor can adsorb on −SH surface sites.^52^ The density of −SH groups, and consequently GPC, would be expected to increase with a higher H_2_ flow ratio, as observed.

Second, increased roughness at higher H_2_ flow ratios and deposition temperatures results in an increase of surface area as well as the amount of reactive fin edges. This, in turn, leads to an increase in GPC with increasing number of ALD cycles due to fin formation and growth. Such an increase of GPC with increasing film thickness has been observed for crystalline MoS_2_ films deposited at ≥300 °C with a low H_2_ flow ratio of 0.20 (ref (36)) as well as other ALD processes depositing crystalline TMDCs.^52,53,68^

As a result of the two mechanisms, the highest number of Mo atoms deposited per cycle, 2.2 at/nm^2^/cycle, was observed using a H_2_ flow ratio of 0.80. It is worth noting that the optically measured GPC conceals changes in film density and stoichiometry, but combining SE with RBS and XPS data suggests that changes in the adsorption density of Mo precursor trumps the density and stoichiometry changes in explaining the GPC trends. In contrast to the surface chemistry and morphology, crystallization itself seems to have at most a moderate effect on GPC compared to the H_2_ flow ratio and morphology. This can be seen comparing H_2_ flow ratios of 0.35 and 0.50 at 250 °C, for example, which exhibit nearly equal GPCs despite the former producing amorphous and the latter crystalline films (Figure S11 in Supporting Information).

### Electrical properties

**Figure 8:**
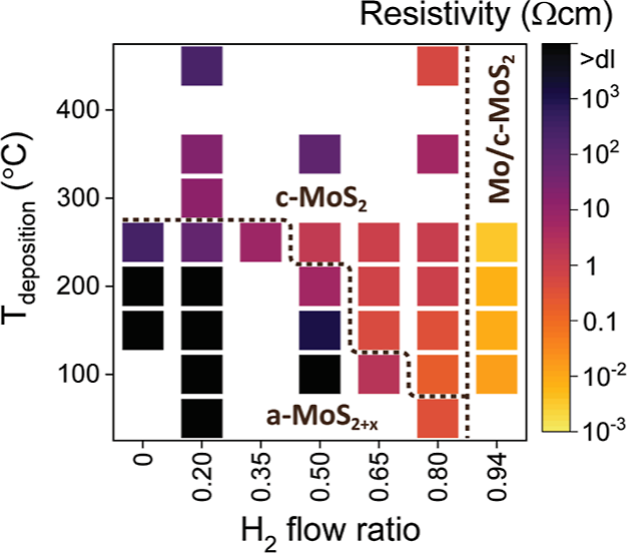
Effect of the H_2_ flow ratio on electrical properties. Heat map of film resistivity as a function of deposition temperature and the H_2_ flow ratio. The films were deposited using 100–140 ALD cycles.

Electrical properties of MoS_2_ play a crucial role in many of its potential applications. We found that the H_2_ flow ratio can be used to control resistivity over several orders of magnitude. At a given temperature, increasing the H_2_ flow ratio led to a decrease in resistivity when a fixed amount of ALD cycles was applied as illustrated in Figure 8 (resistivity values along with other film characteristics can be found in Table S2 in Supporting Information). For amorphous films, the resistivity changes can be correlated to stoichiometry. The most S-rich films (S/Mo ≥ 3) had very high resistivities exceeding the detection limit of our four-point probe instrument (on the order of 1000 Ω cm for 10 nm thick films). A decrease in the S/Mo ratio as a result of increasing the H_2_ flow ratio and/or deposition temperature led to a decrease in resistivity such that the lowest resistivity for an amorphous film (2 Ω cm) was obtained for a-MoS_2.2_ films deposited at 100 °C using a H_2_ flow ratio of 0.65.

For crystalline films, increasing the H_2_ flow ratio at a given temperature resulted in a decrease of resistivity. The lowest resistivities for c-MoS_2_, which were on the order of 0.1–1 Ω cm, were reached using a H_2_ flow ratio of 0.80. These values are much lower compared to the crystalline films grown using a low H_2_ flow ratio of 0.20 at ≥300 °C, namely 15 Ω cm (300 °C) to 220 Ω cm (450 °C). Using a H_2_ flow ratio of 0.94, resistivities as low as 4 mΩ cm were measured, which is likely due to the presence of highly conductive Mo phase in the films. Regarding temperature, the lowest resistivities for c-MoS_2_ were achieved at the lowest temperatures of 100–150 °C.

AC Hall effect measurements were performed to understand the origin of the changes in resistivity. Modest Hall mobilities on the order of 0.001–0.01 cm^2^ V^–1^ s^–1^ and high majority carrier (hole) concentrations on the order of 10^21^ cm^–3^ were measured for c-MoS_2_ films deposited using H_2_ flow ratios of 0.50–0.80 at temperatures of 100–250 °C (data presented and discussed in more detail in Section S10 in Supporting Information). For comparison, reference c-MoS_2_ films deposited at elevated temperatures of 350–450 °C using a low H_2_ flow ratio of 0.20 exhibited significantly lower carrier densities on the order of 10^17^ to 10^18^ cm^–3^ and comparable or slightly higher mobilities on the order of 0.01 cm^2^ V^–1^ s^–1^ as measured here and by Vandalon et al.^69^ Similar mobilities at 150 and 450 °C suggest comparable electronic quality at these rather different deposition temperatures. The high carrier concentrations—as well as mobilities—of our low-temperature c-MoS_2_ films are comparable to MoS_2_ films doped with 10 at. % Al at a deposition temperature of 450 °C as reported by Vandalon et al.^69^ Thus, our process provides a simple, low-temperature route to highly doped p-type MoS_2_ films.

Looking beyond the low-*T* c-MoS_2_ films, we note that increasing the H_2_ flow ratio from 0.20 to 0.80 at 450 °C increased the carrier concentration by 3 orders of magnitude (10^17^ to 10^20^ cm^–3^), showing that the H_2_ flow ratio can be used to control the carrier concentration and resistivity also at higher deposition temperatures. Finally, applying the highest H_2_ flow ratio of 0.94 resulted in extremely high carrier concentrations on the order of 10^22^ cm^–3^, which approaches that reported for Mo thin films (5 × 10^22^ to 1 × 10^23^ cm^–3^)^70,71^ in line with the films partially consisting of Mo.

Considering that our c-MoS_2_ films were rather close to stoichiometric (MoS_1.8_–MoS_2.2_ by XPS) and H was identified as the main impurity by RBS, we believe that the high carrier concentrations are largely due to the incorporated H. For example, 12 at. % H found in the film grown at 150 °C using a H_2_ flow ratio of 0.65 translates to 6 × 10^21^ H atoms/cm^3^, three times higher than the measured carrier concentration of this film. Although the role of N and O impurities on electrical properties cannot be ruled out, the concentration of these elements is significantly lower and also lower than the carrier concentration. Besides impurities, other defects including vacancies (S or Mo) and grain boundaries may contribute to the high carrier density.

Turning back to H, different ways for it to bond in MoS_2_ and its effects on electrical properties have been discussed in the literature. Experimentally, it is very difficult to locate where and how H is bonded. Therefore, this aspect has been mostly studied by DFT calculations, which in some cases have been used to explain the observed electrical properties of MoS_2_ films incorporating hydrogen. Briefly, H has been suggested to bind to S vacancies often present in MoS_2_ (experimentally resulting in either n-type^72^ or p-type^73^ doping), or to S forming thiol (−S–H) groups (DFT suggesting n-type^74,75^ doping), or be located in interstitial sites in the Mo plane (n-type^75,76^ doping by DFT). Intercalated hydrogen in the form of H_2_ is reported to be electrically neutral by DFT^74^ and would be expected to be easily removed from the films in practice. In contrast, we see no major change in electrical properties upon annealing up to 300 °C (Section S12 in Supporting Information). Recently, it has been suggested that H could even replace S in MoS_2_ structure, resulting in n-type MoSH.^77^

Our efforts to locate H using infrared and Raman spectroscopy measurements were unsuccessful, as no evidence of either Mo–H or S–H bonding was observed. However, this is not evidence for the absence of such bonding. In chemical terms, taking into account our deposition process and the detected S deficiency for the crystalline films grown using elevated H_2_ flow ratios, two of the above possibilities seem most likely. The first is binding of H to S vacancies, the presence of which can be inferred from film stoichiometry, and which according to reported experiments can result in either n-type or p-type doping. The second one is thiol groups, which we have speculated to be important for film growth. Furthermore, the thiol group density is expected to increase with increasing H_2_ flow ratio, which is in line with the observed increase of doping level.

Field-effect transistor (FET) devices were constructed to further study the electrical properties of our low-temperature MoS_2_ films. For this purpose, an approximately 5 nm film was prepared at the lowest deposition temperature of 100 °C using a H_2_ flow ratio of 0.80. A reference film of similar thickness was prepared at 350 °C using a low H_2_ flow ratio of 0.20. Both samples exhibited p-type conduction with current on/off ratios of approximately 3–4 (Figure S13 in Supporting Information). P-type conduction is promising as few p-type 2D semiconductors are known in contrast to numerous n-type materials.^78^ The output current of the 100 °C FET was more than an order of magnitude higher compared to the 350 °C reference, in line with the lower four-point probe resistivity and higher carrier concentration of the 100 °C sample. The contacts of the 100 °C FET exhibited clearly non-linear *I*–*V* behavior suggesting presence of a Schottky barrier, whereas the contacts of the 350 °C sample were found to be close to Ohmic.

Cross-sectional TEM images of devices fabricated from the two samples revealed an approximately 2–3 nm thick amorphous interface layer between MoS_2_ and Ni contacts for the 100 °C sample compared to a thinner, approximately 1 nm interface layer for the 350 °C sample curves (Figure S14 in Supporting Information). The thicker interface layer of the 100 °C sample may explain the observed non-linear *I*–*V*. Furthermore, we found that the films deposited at 100 °C began to oxidize when annealed in air at temperatures as low as 150 °C, which should be taken into account during device processing (see discussion in Sections S11 and S12 in Supporting Information). Thus, besides further optimization of the deposition conditions to control carrier density, evaluation of contact/MoS_2_ interfaces and MoS_2_ stability during processing are important topics for further studies on low-temperature PEALD MoS_2_.

### Applying the Concept to Other TMDCs (TiS_2_ and WS_2_)

Besides MoS_2_, we found that the H_2_ flow ratio is an important parameter for low-temperature PEALD of TMDCs in general, enabling deposition of several materials in crystalline form at lower temperatures than has been achieved so far. Furthermore, the concept is likely also applicable to other sulfides grown by PEALD and may, for example, enable controlling the phase of metal sulfides that exist in different stoichiometries. Here, we look at PEALD processes developed earlier in our group for semiconducting WS_2_ and metallic TiS_2_.^52,53^ Both reported processes use H_2_S/Ar plasma as the reactant without any H_2_ (H_2_ flow ratio of 0).

**Figure 9:**
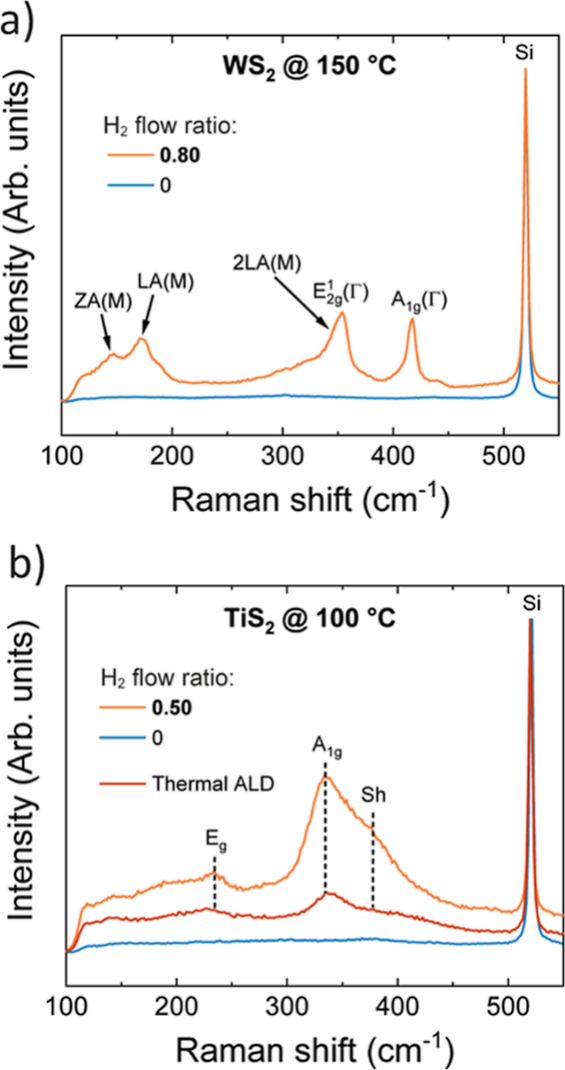
Effect of the H_2_ flow ratio on the growth of other TMDCs at low temperatures. Raman spectra of (a) WS_*x*_ (150 °C) and (b) TiS_*x*_ (100 °C) films grown using different H_2_ flow ratios. The peaks have been labeled based on refs (82)–^84^ (Sh = shoulder, a TiS_2_ mode commonly observed but has not been conclusively identified in the literature).

The lowest temperature at which the tungsten sulfide process produces crystalline WS_2_ films is 300 °C.^52,67,79^ We found that films deposited at 150 °C without H_2_ were highly S-rich (WS_4.0_ by XPS), amorphous, and highly resistive similar to the MoS_2_ films deposited under analogous conditions. Increasing the H_2_ flow ratio to 0.80 at 150 °C resulted in crystalline WS_2.1_ films with a resistivity of 4 Ω cm (Raman spectra in Figure 9a, other characterization in Section S13 in Supporting Information). The similarity between MoS_2_ and WS_2_ is understandable considering Mo and W are in the same group in the periodic table and the fact that both processes use analogous M(N^*t*^Bu)_2_(NMe_2_)_2_ precursors (M = Mo, W).

The only TiS_2_ PEALD process reported in the literature produces crystalline films at 150 °C and above, while at 100 °C amorphous TiS_3_ films are deposited.^53^ Besides this PEALD process, a thermal TiS_2_ process using the same titanium precursor Ti(NMe_2_)_4_ has been reported.^80,81^ The thermal process deposits crystalline TiS_2_ films at temperatures as low as 100 °C, which suggests that the inability of the PEALD process to deposit crystalline films at 100 °C is due to incorporation of excess S in the films rather than insufficient energy for crystallization, for example. Indeed, modifying the reported PEALD process to use a H_2_ flow ratio of 0.50, we were able to deposit TiS_2_ films at 100 °C with improved crystallinity (Figure 9b) and stoichiometry (Table S7 in Supporting Information) over the reported thermal process. In the case of TiS_2_, the H_2_ flow ratio of 0.80 used for MoS_2_ and WS_2_ was found to be too high, resulting in S-poor films (not shown).

### Insights into the Role(s) of Hydrogen in Film Growth

So far in this article, we have demonstrated that the addition of H_2_ into H_2_S/Ar plasma feed gas controls stoichiometry, crystallinity, film growth, morphology, and electrical properties of PEALD TMDC films in conjunction with deposition temperature. In this section as well as a schematic (Figure S19) and supplementary discussion (Section S14) provided in Supporting Information, we describe our understanding on the roles that hydrogen plays in the deposition process along with other process variables.

Optical emission spectroscopy (OES)^85^ was used to investigate species created in H_2_S/H_2_/Ar plasma with varying H_2_ flow ratios. The measurements suggest that different S containing species including S, S^+^, S_2_, SH^+^, and H_2_S^+^ are created, while for hydrogen the main reactive species is atomic hydrogen, that is, H radicals (Figure S20 in Supporting Information). Differentiating between different sulfur species is difficult by OES, making potential changes in types of plasma species as a function of the H_2_ flow ratio challenging to infer. Observations on the balance between S and H species can be made, however. In our experiments, the total flow of H_2_S and H_2_ was fixed to 10 sccm. Thus, the observed increase in emission intensity of atomic H and the simultaneous decrease of reactive sulfur species with increasing H_2_ flow ratio is in line with changes in flow rates (Figure S21 in Supporting Information). It is worth noting that H_2_S contains hydrogen, so some atomic H is produced even when no H_2_ is added.

An increase in the ratio of atomic H to reactive sulfur species can affect the deposition chemistry, either by preventing excess sulfur incorporation in the first place, or by introducing a competing reaction that removes S. While the first possibility is challenging to confirm, we performed an additional experiment to probe the ability of H to remove excess S. Namely, we added a separate H_2_ gas or H_2_ plasma step after a H_2_S/H_2_/Ar plasma step (H_2_ flow ratio of 0.20) at 150 °C—plasma conditions that would otherwise produce a-MoS_3.4_ films. The results confirmed that H_2_ plasma is capable of removing the extra S, thus resulting in c-MoS_2_ films, whereas H_2_ gas did not affect stoichiometry or crystallinity (Table S8 in Supporting Information). Although this observation does not exclude the first scenario, that is, the possibility of an elevated H_2_ flow ratio to prevent incorporation of extra S in the first place, it does prove that H radicals can turn a-MoS_2+*x*_ into c-MoS_2_ under our ALD conditions.

Additional deposition experiments explored the effect of the H_2_S flow rate, which was found to be an important parameter besides H_2_ flow ratio and deposition temperature. Decreasing the H_2_S flow ratio resulted in less S incorporation similar to an increase of the H_2_ flow ratio. In this way, crystalline films could be achieved at 250 °C without any added H_2_ (Figure S22a in Supporting Information). At lower temperatures, such as 150 °C, however, addition of H_2_ was found necessary in order to deposit c-MoS_2_ films (Figure S22b in Supporting Information). Furthermore, at a fixed H_2_ flow ratio, an increase of the H_2_S flow rate increased S incorporation. Thus, in practice both the H_2_ flow ratio and absolute flow rates of H_2_ and H_2_S, as well as deposition temperature, must be considered to achieve desired film properties.

## Conclusions

We have demonstrated a PEALD process capable of deposition of wafer-scale polycrystalline TMDC films, including MoS_2_, TiS_2_, and WS_2_, of accurately controlled thickness at record-low temperatures down to 100 °C. Compared to earlier PEALD processes, we were able to substantially lower the deposition temperature by controlling the amount of H_2_ added into plasma feed gas, which prevents incorporation of excess S and thus enables crystallization of the deposited films. Systematic characterization of film growth and properties as a function of deposition temperature and the H_2_ flow ratio revealed the interplay of these two parameters on stoichiometry, crystallinity, growth per cycle, morphology, and electrical properties. The identification and quantification of these parameters also enables further development of PEALD for low-temperature growth of crystalline chalcogenides of controlled stoichiometry. In terms of electrical properties, we found that low-temperature MoS_2_ films had low resistivities of 0.1–1 Ω cm. Hall measurements revealed that the films exhibited p-type conduction with high carrier concentrations reaching up to 10^21^ cm^–3^. We attribute the high hole concentrations mainly to incorporation of H from the plasma. MoS_2_ deposited at 100 °C was evaluated as a channel material for FETs, which together with ability to deposit films on flexible and transparent PET substrates paves way to applications in flexible electronics and other fields requiring low deposition temperatures.

## Experimental Section

### Film Deposition

MoS_*x*_ thin films were deposited using an Oxford Instruments FlexAL PEALD reactor equipped with a 13.56 MHz remote inductively coupled plasma (ICP) source. The chamber is pumped with a turbomolecular pump capable of reaching a base pressure of 10^–6^ Torr. The reactor walls can be heated up to 150 °C. Thus, for depositions at 50–150 °C, the wall and substrate temperatures were set to the same value. For deposition temperatures of 200–450 °C, the substrate table was heated to the indicated temperature, while reactor walls were at 150 °C. Due to limited thermal contact between the table and the substrate at the low operating pressures (see below), the actual substrate temperature was lower than the table temperature.^86^ For consistency within the article and preceding literature, table temperatures are used throughout this article. At temperatures mostly discussed in this article, 150 °C and below, the difference in actual and set temperatures is negligible.

Silicon (100) substrates with a 450 nm thermally grown SiO_2_ layer on top to enhance optical contrast (Siegert Wafer) were used for most of the depositions. Additionally, fused SiO_2_ substrates were used for some of the Hall measurements and Si substrates with 90 nm of thermal SiO_2_ was used for the transistor characterization. PET foil (Melinex ST504 from DuPont Teijin Films, 125 μm thickness) was used to demonstrate capability to deposit MoS_2_ on plastics at low temperatures. Both uncoated and ALD Al_2_O_3_-coated (50 ALD cycles using AlMe_3_ and H_2_O) PET substrates yielded similar results.

The substrates were used as received apart from cutting to desired size and blowing clean with pressurized N_2_. The substrates with typical size ranging from 1 × 1 to 3 × 3 cm^2^ were loaded on an 8″ carrier wafer, which was introduced into a load lock and evacuated to less than 10^–2^ Torr before introduction into the reaction chamber. The substrates were allowed to equilibrate to the chamber temperature for 10 min under Ar flow at 200 mTorr before the deposition was started.

The MoS_2_ ALD process is based on the work of Sharma et al.,^36^ the main difference being the modified plasma feed gas composition. The total flow of H_2_S (99.5% purity), H_2_ (99.999%), and Ar (99.999%) gases (all supplied by Linde Gas) through the ICP tube during the plasma pulse was kept constant at 50 sccm, resulting in approximately 6 mTorr pressure in the chamber. The experiments focused on varying the flow rates of H_2_ and H_2_S, which were kept at a total of 10 sccm except for the highest H_2_ flow ratio of 0.94. The gas flows and the respective H_2_ flow ratios, which are used to describe the flow rates used throughout the article, are listed in Table 2. In the preceding work of Sharma et al.,^36^ H_2_S and H_2_ flow rates were 8 and 2 sccm (not 2 and 8 sccm as erroneously stated in the article), corresponding to a H_2_ flow ratio of 0.20.

**Table 2:** Gas Flows through the ICP Tube for Different H_2_ Flow Ratios^a^

H_2_ flow ratio H_2_/(H_2_ + H_2_S)	H_2_S flow rate (sccm)	H_2_ flow rate (sccm)	Ar flow rate (sccm)
0	10	0	40
0.20	8	2	40
0.35	6.5	3.5	40
0.50	5	5	40
0.65	3.5	6.5	40
0.80	2	8	40
0.94	2	30	18

a In each case, the total gas flow was 50 sccm.

An ALD cycle consisted of four main steps: (1) 6 s dose of Mo(N^*t*^Bu)_2_(NMe_2_)_2_ precursor, (2) purging for 10 s (of which 6 s with 300 sccm Ar flown into the chamber and 4 s pumping the chamber with no flow), (3) H_2_S/H_2_/Ar plasma for 20 s (100 W ICP power), and (4) purging for 6 s with 300 sccm Ar flow. Mo(N^*t*^Bu)_2_(NMe_2_)_2_ (98%, Strem Chemicals) was heated to 50 °C in an external canister and supplied with 50 sccm Ar flow through delivery lines that were heated to 70 °C to prevent condensation. Additional, 4 and 5 s long pressure and gas flow stabilization steps preceded the Mo precursor and plasma pulses, respectively. Thus, the total length of one ALD cycle was 51 s. The chamber pressure was set to 200 mTorr during the Mo(N^*t*^Bu)_2_(NMe_2_)_2_ pulse using an automatic pressure control valve. During the plasma pulse, pressure was approximately 6 mTorr with the automatic pressure control valve open as a result of the pumping speed and the gas flows used.

Unless otherwise noted, the number of ALD cycles was in most of the depositions either 100 (450 °C), 120 (50 °C, 300 °C, and 350 °C), or 140 (100–250 °C). This number of cycles was chosen to result in approximately 10 nm thick films at each temperature for a H_2_ flow ratio of 0.20. For 50–100 °C, this information was not available. To warrant these lowest temperatures being in the ALD regime, it was verified that the H_2_S/H_2_/Ar plasma does not result in S deposition by itself using SE (data not shown) and that the Mo precursor does not condense on the substrate.^87^

Tungsten sulfide^52^ and titanium sulfide^53^ depositions were performed following literature procedures. The only and crucial modification was the plasma feed gas composition (H_2_ flow ratio). Both published processes used a H_2_ flow ratio of 0 and produced highly S-rich films at low temperatures. In this work, WS_2_ was deposited at 150 °C using a H_2_ flow ratio of 0.80 and TiS_2_ at 100 °C using a H_2_ flow ratio of 0.50. A thermal TiS_*x*_ recipe was also used for comparison.^80^

### Film Characterization

Film thickness was monitored during depositions using in situ spectroscopic ellipsometry (SE). Either an M-2000FI (spectral range 0.8–5.0 eV; typically 0.8–4.0 eV used for fitting) or M-2000F instrument (spectral range 1.2–5.0 eV) by J.A. Woollam was used. Typically, a measurement was performed every 10 ALD cycles. Thicknesses were extracted using a MoS_*x*_/SiO_2_/interlayer/Si model in CompleteEASE 5.1 software. MoS_*x*_ was modeled using a B-spline layer, whereas for other layers optical constants provided with the software were used. For crystalline MoS_2_ films, surface roughness was accounted for using effective medium approximation with 50% void content. To approximate the amount of material deposited, the reported thicknesses consist of the thickness of the dense MoS_*x*_ layer plus half the thickness of the surface roughness layer.

For a typical fitting procedure, the SiO_2_ thickness and substrate temperature were first extracted from a spectrum measured immediately before the first ALD cycle. The thickness and optical constants of the MoS_*x*_ layer were fitted for the last spectrum measured immediately after the last ALD cycle. For the other spectra measured in between these two extreme points, only the MoS_*x*_ layer thickness (and possible surface roughness) were fitted. The reported thicknesses represent averages of numerous grains with slightly different thicknesses and optical properties in the analysis area of a few mm^2^.

Ex situ thickness measurements were also done for selected samples using a J.A. Woollam M-2000U instrument equipped with a variable angle stage. Data were acquired in the spectral range of 1.2–6.5 eV using 65, 75, and 85° angles with respect to surface normal. The data were fit for thickness of both MoS_2_ and SiO_2_ typically using fixed optical constants determined previously by in situ measurements. Ex situ uniformity mapping was performed using a J.A. Woollam M-2000 instrument (spectral range 1.2–3.3 eV) equipped with an automated mapping stage. Mapping was done in a circular pattern excluding areas close to wafer edges. Besides a 5 nm thick MoS_2_ film on a 450 nm SiO_2_/Si wafer (4″), a bare 450 nm SiO_2_/Si substrate was also measured for reference. The data were fit for thickness of both MoS_2_ and SiO_2_ with fixed optical constants.

Sheet resistance of the films was measured using a four-point probe (consisting of a Keithley 2400 SourceMeter and a Signatone SP4-40045TRS probe head). Film resistivity was calculated by multiplying the sheet resistance by the film thickness measured by in situ SE. Hall effect measurements were performed in van der Pauw geometry on approximately 1 × 1 cm^2^ samples to extract carrier mobility and concentration (Lakeshore 8404 HMS). Both SiO_2_/Si and fused SiO_2_ substrates were used for the Hall measurements, the latter having the advantage of not requiring cutting after deposition as current leakage to conductive Si via film deposited on the sides of the substrate was not possible. AC Hall measurements were used to better detect the low Hall voltages resulting from low mobilities and high carrier concentrations. Measurements were repeated multiple times (without removing the sample) to evaluate measurement uncertainty.

Film morphology was examined by SEM (Zeiss Sigma) using an acceleration voltage of 3 kV and an InLens detector. Morphology and surface roughness were analyzed by AFM (Bruker Dimension Icon) in PeakForce Tapping based ScanAsyst mode in air. Probes with a nominal spring constant of 0.4 N/m and a tip radius of 2 nm were used (ScanAsyst-air). Roughness was determined as root-mean-square (*R*_q_) value after flattening the 500 × 500 nm^2^ image (1st order) using Bruker Nanoscope 2.0 software.

Film crystallinity was evaluated by Raman spectroscopy using a confocal Raman microscope (Renishaw inVia) with a 514 nm laser, 50× objective (NA 0.75), and 1800 lines/mm grating. Six spectra of 10 s each were accumulated during each measurement. Laser power was estimated as 0.6 mW on the sample (100 mW laser with 1% neutral density filter).

XPS was used for stoichiometry measurements and analysis of chemical environment using a Thermo Scientific K-Alpha spectrometer equipped with a monochromatic Al Kα source (*h*ν = 1486.6 eV) focused to 400 μm diameter spot on the sample. An electron flood gun was used to minimize charging effects during measurements. The measured spectra were referenced to a binding energy of 284.8 eV for C 1s peak of adventitious carbon. Pass energies of 50 and 20 eV were used for survey and high-resolution spectra. Avantage software was employed for peak deconvolution using the Gaussian–Lorentzian sum function to describe the individual components and a Shirley-type background. Additional XPS depth-profiling experiments (results not shown) were performed for selected samples to ensure no major carbon contamination was present. Sputtering was done using Ar^+^ ions (500 eV) and a spectrum was measured after every 30 s of sputtering.

For selected samples, RBS and ERD measurements were performed by Detect 99 B.V. (Eindhoven, The Netherlands) using a 2 MeV He^+^ beam. RBS was used to determine absolute atom density, film stoichiometry, and impurities besides hydrogen, while ERD was used to determine hydrogen concentration in the films. The reported uncertainties take into account known systematic and statistical uncertainties. Mass density of the films was calculated using RBS atom densities and SE thicknesses.

The cross-sectional TEM samples were prepared by FEI Helios Nanolab 600 or 600i dual-beam focused-ion beam FIB/SEM instruments following a standard lift-out procedure. Before ion milling, C/Pt or SiO_2_ mask layers were deposited in the dual-beam instruments. The TEM imaging was performed using a probe-corrected JEOL ARM 200F instrument operated at 200 kV. The thick (∼50 nm) 150 °C sample was used for sample preparation as-deposited, while the thin (∼5 nm) 100 and 350 °C samples went through a transistor channel patterning and Au/Ni metal deposition procedure before FIB milling of the TEM samples.

## Supporting Information Available

Process saturation curves, full sets of characterization data and additional discussion (AFM, Raman, SE, SEM, TEM, XPS, XRD), discussion of Hall measurements and results, output data and discussion of FETs and MoS_2_/Ni contacts, results of thermal stability (annealing) studies, characterization of WS_*x*_ and TiS_*x*_ films, and additional experiments and discussion on roles of H_2_ and H_2_S (PDF)
