# Supplementary material for: Atomic Layer Deposition of Large-Area Polycrystalline Transition Metal Dichalcogenides from 100 °C through Control of Plasma Chemistry
Source: Chem Mater. 2022 Aug 5;34(16):7280–92. doi: 10.1021/acs.chemmater.2c01154 (PMC9404538; doi:10.1021/acs.chemmater.2c01154)
Supplement: Supplementary file 1 — cm2c01154_si_001.pdf [file cm2c01154_si_001.pdf]

Supporting information

Atomic Layer Deposition of Large-Area Polycrystalline Transition Metal Dichalcogenides from 100 °C through Control of Plasma Chemistry

Miika Mattinen,[1] Farzan Gity,[2] Emma Coleman,[2] Joris F. A. Vonk,[1] Marcel A. Verheijen,[1,3] Ray Duffy,[2] Wilhelmus M. M. Kessels,[1] and Ageeth A. Bol*[1,4]

1 Department of Applied Physics, Eindhoven University of Technology, PO Box 513, 5600 MB Eindhoven, The Netherlands

2 Tyndall National Institute, University College Cork, Lee Maltings, Dyke Parade, Cork T12 R5CP, Ireland

3 Eurofins Materials Science Netherlands, High Tech Campus 11, 5656 AE Eindhoven, The Netherlands

4 Department of Chemistry, University of Michigan, 930 N. University Ave, Ann Arbor, MI, 48109-1055, United States of America

*corresponding author: a.a.bol@tue.nl

Contents

S1. Saturation of surface reactions S2

S2. X-ray diffraction S4

S3. AFM images of few-layer MoS_2 films S5

S4. Raman spectra S6

S5. XPS measurements S7

S6. SEM images S11

S7. Cross-sectional TEM images before and after filtering S12

S8. In situ spectroscopic ellipsometry S13

S9. Tabulated data of prepared samples and their characteristics S15

S10. Hall measurements S17

S11. Field effect transistors S19

S12. Mild annealing of MoS_2 deposited at low temperatures S22

S13. Other TMDCs: TiS_x and WS_x S25

S14. Insight into the roles of hydrogen (and process conditions) S26

S15. References S32

S1. Saturation of surface reactions

Saturation of surface reactions, a hallmark of ALD, can be tested by varying precursor doses and measuring their effect on the thickness increase per ALD cycle, called growth per cycle (GPC). For a proper ALD process, the GPC stops increasing (saturates) after a sufficient precursor dose is provided, which indicates that all reactive surface sites are consumed and no parasitic reactions are occurring. The results of such experiments are shown in Figure S1 for two H_2 flow ratios at a temperature of 150 °C: low (0.20) and high (0.80), resulting in amorphous MoS_{2+x} and crystalline MoS_2 films, respectively. The high H_2 flow ratio leads to considerably higher GPC due to the crystallinity and morphology differences as discussed in the article.

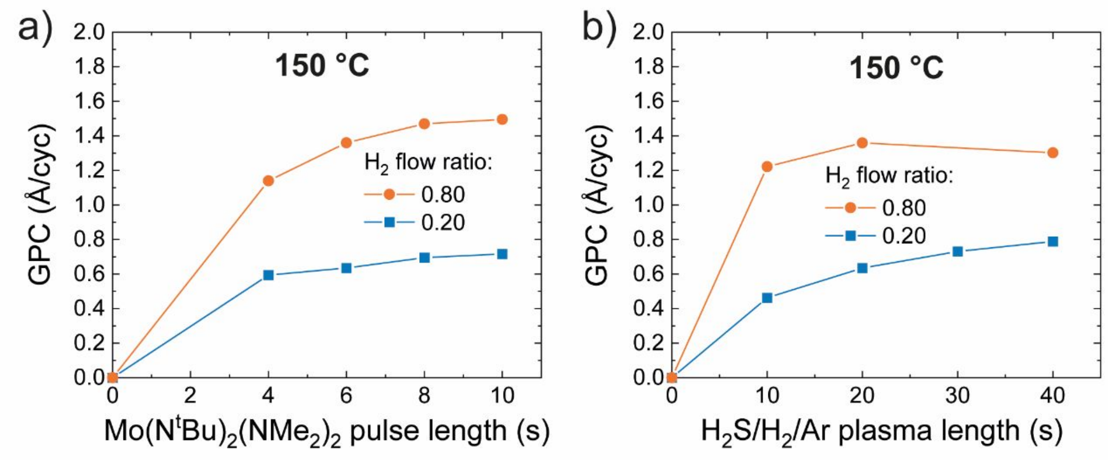

Figure S1. Saturation curves showing GPC as a function of a) $Mo(N^tBu)_2(NMe_2)_2$ and b) $H_2S/H_2/Ar$ plasma pulse length at 150 °C for two H_2 flow ratios (0.2 and 0.8). In a), $H_2S/H_2/Ar$ plasma pulse length was fixed at 20 s, while in b) the $Mo(N^tBu)_2(NMe_2)_2$ pulse length was 6 s.

For the amorphous films that grow in linear fashion (H_2 flow ratio of 0.20), an in situ approach was used. In this approach, the film thickness was measured after every cycle by in situ SE and the measured GPCs were averaged over 20 consecutive cycles, after which one pulse length was varied and the procedure was repeated until all of the pulse times had been tested. Thus, all the data using an H_2 flow ratio of 0.20 were acquired during a single deposition. To ensure the reliability of in situ data collection, it was confirmed that the growth rate at the beginning and end of the deposition were identical using the same pulse lengths. The GPC reached a value of approximately 0.6 Å/cycle using 4 s $Mo(N^tBu)_2(NMe_2)_2$ pulses with a slight increase upon further increase of pulse length. Similarly, increasing $H_2S/H_2/Ar$ plasma exposure beyond the standard 20 s resulted in a slight increase of GPC, reaching nearly 0.8 Å/cycle at 40 s plasma exposure. Such a "soft saturation" behavior may be due to slow surface reactions or changes in the structure of the deposited amorphous film. Separate experiments confirmed that the S/Mo stoichiometry of the deposited films did not change upon an increase of $H_2S/H_2/Ar$ plasma exposure from 10 to 40 s, however. Other typical causes for increasing GPC such as precursor decomposition or condensation could also be ruled out at this temperature (see Experimental section in the article and Ref.[1,2]).

For the crystalline films (H_2 flow ratio of 0.80), an ex-situ saturation curve approach was used to produce the data in Figure S1, where each data point represents an average GPC from a separate 100 cycle deposition experiment (GPC = final thickness / 100). The use of the in situ approach described above was prevented by the increase of GPC with increasing number of ALD cycles, which results from rough morphology as discussed in the article. Saturation at 1.5 Å/cycle was reached using 8 s $Mo(N^tBu)_2(NMe_2)_2$ and 20 s $H_2S/H_2/Ar$ plasma exposures.

For further experiments, pulse lengths of 6 s for $Mo(N^tBu)_2(NMe_2)_2$ and 20 s for $H_2S/H_2/Ar$ plasma were chosen to be used in the more than 30 different conditions (combination of deposition temperature and H_2 flow ratio) evaluated in this work. The choice is also in line with the work of Sharma et al.[1] who found these pulse lengths to be sufficient for saturation at higher temperatures of 250 and 450 °C (H_2 flow ratio of 0.20). Furthermore, good film uniformity and repeatability was confirmed even if the pulses were in slight undersaturation for some conditions, such as H_2 flow ratio of 0.80 at 150 °C (Fig 1c).

S2. X-ray diffraction

For a complementary analysis of film crystallinity besides Raman spectroscopy and cross-sectional TEM, grazing incidence (ω = 0.4°) XRD measurements were performed on films deposited at 150 °C using different H_2 flow ratios. The measurements were done using a Bruker D8 Advance diffractometer equipped with a Cu Kα (λ = 1.54 Å) X-ray tube. A relatively broad peak attributed to the (002) reflection of MoS_2 (i.e. the 2D basal planes) was observed for films deposited using H_2 flow ratios of 0.65, 0.80, and 0.94 (Figure S2). The broad, weaker peak observed at a similar position for H_2 flow ratio of 0.50 is attributed to short-range order of a-MoS_3 type material.[3–5] The broadness of the peaks is a result of nanocrystallinity and low film thickness. Furthermore, a shift to smaller angles, i.e. larger lattice spacing is obvious especially for the highest H_2 flow ratios of 0.80 and 0.94, which may be due to hydrogen incorporated into the films (see section *Film composition* in the article). For the H_2 flow ratio of 0.80, a weak additional peak attributed to (100) or (101) planes was also observed, in line with the largest roughness of this film and thus the presence of a large portion of crystallites oriented with their basal planes non-parallel to the substrates. A very broad peak at approximately 2θ = 22° is a result of the short-range order in the amorphous thermal SiO_2 layer of the SiO_2/Si substrate.

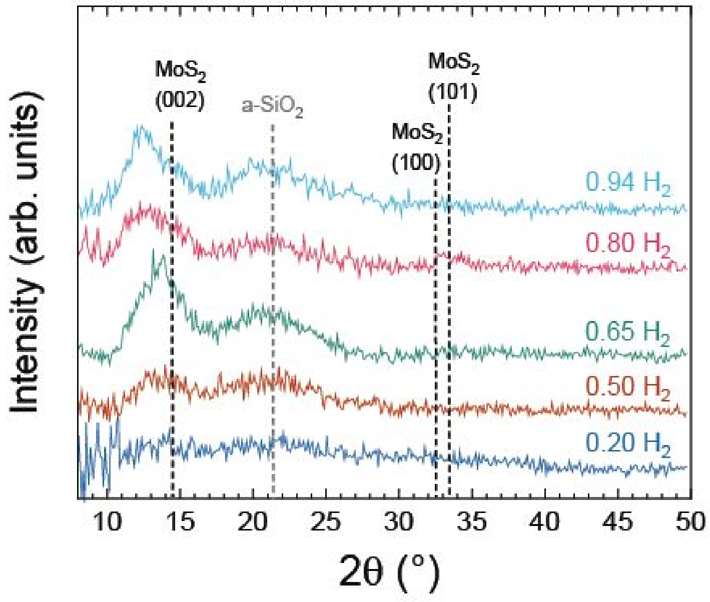

Figure S2. Grazing incidence X-ray diffractograms of MoS_x films deposited at 150 °C (140 ALD cycles) on 450 nm SiO_2/Si substrates using different H_2 flow ratios. Reference positions for selected MoS_2 reflections (JCDPS-ICDD powder diffraction file 00-037-1492) as well as a typical position for amorphous SiO_2 (a-SiO_2) are shown. The data is shown after background removal. The higher noise level below approximately 2θ = 10° is a result of an automatic attenuator used at these lowest angles due to high background resulting from air scattering.

S3. AFM images of few-layer MoS_2 films

AFM images of few-layer films deposited at 150 °C using an H_2 flow ratio of 0.80 show that the films are relatively smooth, although rougher than the SiO_2/Si substrate (Figures S3, S4). The roughness increases with increasing thickness, which can be attributed to the fraction of out-of-plane oriented crystallites increasing with increasing thickness as well as the fact that individual crystallites may grow at slightly different rates. Thus, the films consist of nanocrystallites with different orientations and slightly different thicknesses. At 10–20 nm thickness range the films grown using a high H_2 flow ratio are rough as the surface has then become covered by out-of-plane oriented crystallites (see SEM images in Figure S8).

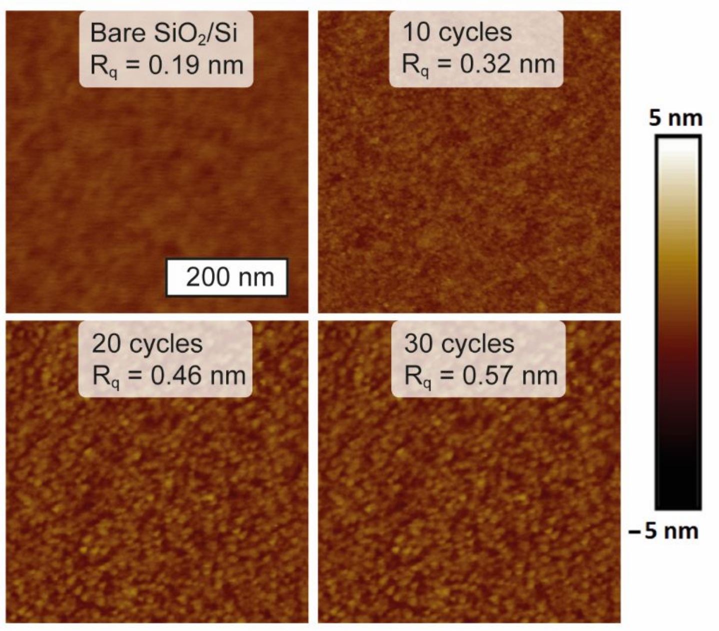

Figure S3. AFM images and roughness values of MoS_2 films deposited using 10 to 30 ALD cycles (150 °C, H_2 flow ratio of 0.80).

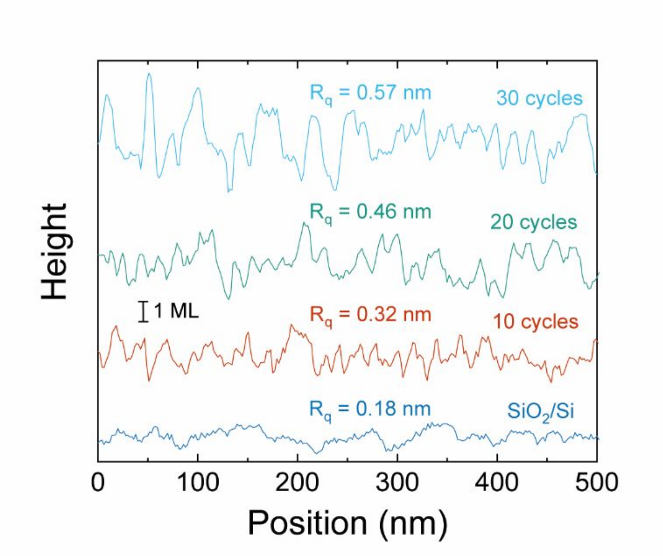

Figure S4. Representative line height profiles extracted from AFM images shown in Figure S3. (150 °C, H_2 flow ratio of 0.80). The lines have been offset vertically for clarity. Approximate thickness of a single MoS_2 monolayer (ML) is shown for comparison.

S4. Raman spectra

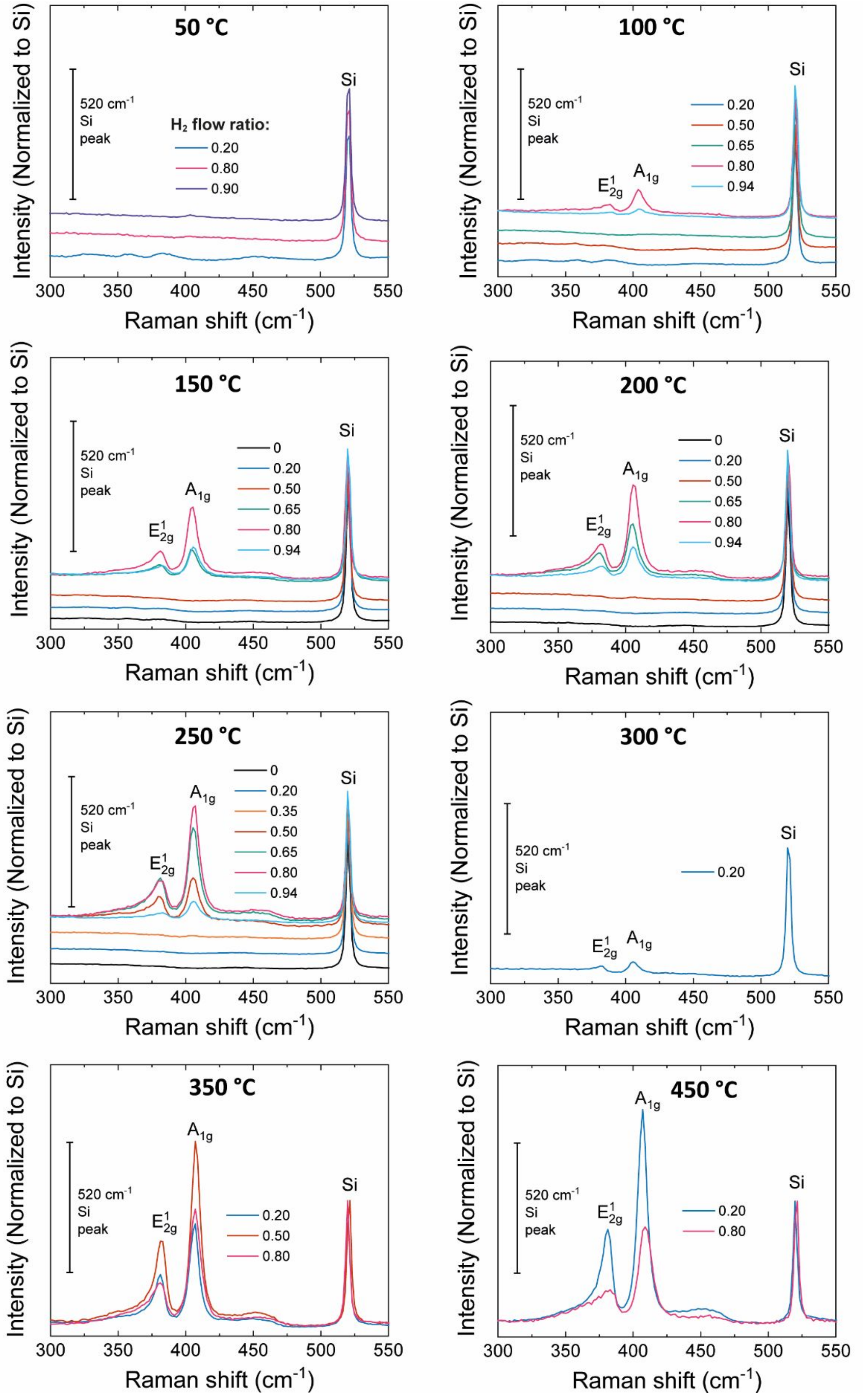

Figure S5. Raman spectra of MoS_x films deposited at different temperatures using different H_2 flow ratios. The number of ALD cycles was 100 (450 °C), 120 (50 °C, 300 °C, and 350 °C), or 140 (100–250 °C). The intensities have been normalized to the Si peak at 520 cm^{-1} to assist comparison of different samples that have different thicknesses. The spectra of amorphous samples have been offset vertically to improve visibility, while the spectra of crystalline films are overlaid for easy comparison.

S5. XPS measurements

Table S1. Results of XPS peak deconvolution for samples deposited at different temperatures using different H_2 flow ratios. The binding energies (BEs) and relative intensities of different components (as defined in the article) for Mo 3d and S 2p regions are listed. The amount the BEs were shifted to place hydrocarbon C 1s BE at 284.8 eV reference position is also indicated (i.e. measured raw BE values can be calculated by deducting this shift from the BEs shown in the table). The films were deposited using 100 (450 °C), 120 (50 °C, 300 °C, and 350 °C), or 140 ALD cycles (100–250 °C)

H_2 flow ratio	S/Mo ratio	Mo $3d_{5/2}$					S $2p_{3/2}$			BE shift to C1s ref.
		Mo^{4+} (MoS_x)	Mo^{5+} (MoO_yS_x)	Mo^{6+} (MoO_3)	$Mo^{(4-\delta)+}$	$Mo(C_xN_y)$	**S^{2-}** & S_2^{2-} (terminal)	**S_2^{2-}** (bridging) & S^{2-} (apical)	S^{2-} (e^- rich)	
**50 °C**										
0.20	7.3	230.0 eV<br>88%	231.0 eV<br>11%	232.4 eV<br>1%	-	-	162.5 eV<br>34%	163.8 eV<br>66%	-	+0.4 eV
0.80	2.0	229.0 eV<br>49%	229.9 eV<br>34%	232.6 eV<br>16%	-	-	162.1 eV<br>84%	163.5 eV<br>16%	-	+0.4 eV
0.90	1.8	229.0 eV<br>52%	230.0 eV<br>30%	232.6 eV<br>18%	-	-	162.2 eV<br>90%	163.8 eV<br>10%	-	+0.5 eV
**100 °C**										
0.20	4.6	229.7 eV<br>63%	230.2 eV<br>31%	232.3 eV<br>5%	-	-	162.8 eV<br>37%	163.7 eV<br>63%	-	+0.3 eV
0.50	3.6	229.5 eV<br>78%	230.0 eV<br>15%	232.3 eV<br>6%	-	-	162.2 eV<br>40%	163.5 eV<br>60%	-	+0.3 eV
0.65	2.2	228.9 eV<br>67%	229.8 eV<br>24%	232.3 eV<br>8%	-	-	161.9 eV<br>76%	163.4 eV<br>24%	-	+0.2 eV
0.80	1.8	229.1 eV<br>80%	230.8 eV<br>6%	232.4 eV<br>10%	228.6 eV<br>3%	-	162.1 eV<br>76%	163.3 eV<br>9%	161.4 eV<br>15%	+0.2 eV
0.94	1.4	228.7 eV<br>45%	230.0 eV<br>16%	232.4 eV<br>11%	-	228.3 eV<br>28%	162.0 eV<br>81%	163.6 eV<br>7%	161.3 eV<br>12%	+0.1 eV
**150 °C**										
0	3.6	229.5 eV<br>70%	230.0 eV<br>22%	232.3 eV<br>6%	-	-	162.3 eV<br>40%	163.5 eV<br>59%	-	+0.4 eV
0.20	3.5	229.4 eV<br>77%	230.0 eV<br>16%	232.3 eV<br>5%	-	-	162.2 eV<br>39%	163.5 eV<br>60%	-	+0.4 eV
0.50	3.0	229.1 eV<br>89%	230.5 eV<br>6%	232.3 eV<br>4%	-	-	161.9 eV<br>49%	163.2 eV<br>51%	-	+0.3 eV
0.65	2.1	229.0 eV<br>76%	230.5 eV<br>9%	232.4 eV<br>7%	228.6 eV<br>7%	-	161.9 eV<br>72%	163.0 eV<br>16%	161.1 eV<br>12%	+0.2 eV
0.80	1.9	229.6 eV<br>79%	231.1 eV<br>4%	232.9 eV<br>9%	228.9 eV<br>3%	-	162.4 eV<br>93%	163.9 eV<br>3%	161.5 eV<br>4%	+0.5 eV
0.94	1.3	229.4 eV<br>55%	231.0 eV<br>7%	232.8 eV<br>12%	-	228.5 eV<br>35%	162.3 eV<br>77%	163.5 eV<br>10%	161.5 eV<br>13%	+0.3 eV
**200 °C**										
0	3.1	229.4 eV<br>77%	230.6 eV<br>15%	232.9 eV<br>7%	-	-	162.1 eV<br>46%	163.4 eV<br>54%	-	+0.3 eV
0.20	3.0	229.4 eV<br>76%	230.2 eV<br>20%	232.2 eV<br>6%	-	-	162.1 eV<br>48%	163.4 eV<br>52%		+0.3 eV

Table S1 continued.

H_2 flow ratio	S/Mo ratio	Mo 3d$_{5/2}$<br>Mo^{4+} (MoS_x)	 Mo^{5+} (MoO_xS_y)	 Mo^{6+} (MoO_3)	 $Mo^{(4-\delta)+}$	 Mo(C_xN_y)	S 2p$_{3/2}$<br>$\mathbf{S^{2-}}$ & S_2^{2-} (terminal)	 $\mathbf{S_2^{2-}}$ (bridging) & S^{2-} (apical)	 S^{2-} (e^- rich)	BE shift to C1s ref.
**200 °C**										
0.50	2.5	229.1 eV<br>73%	230.2 eV<br>20%	232.3 eV<br>6%		-	162.0 eV<br>50%	163.2 eV<br>45%	161.0 eV<br>5%	+0.3 eV
0.65	2.2	229.4 eV<br>76%	230.8 eV<br>10%	232.6 eV<br>8%	228.7 eV<br>6%	-	162.3 eV<br>68%	163.6 eV<br>15%	161.5 eV<br>18%	+0.5 eV
0.80	1.9	229.6 eV<br>68%	230.0 eV<br>16%	232.7 eV<br>9%	228.9 eV<br>7%	-	162.5 eV<br>86%	164.0 eV<br>6%	161.5 eV<br>8%	+0.6 eV
0.94	1.3	229.5 eV<br>27%	230.6 eV<br>9%	232.4 eV<br>11%	-	228.6 eV<br>53%	162.3 eV<br>81%	163.8 eV<br>9%	161.4 eV<br>10%	+0.4 eV
**250 °C**										
0	2.8	229.3 eV<br>79%	230.0 eV<br>17%	232.5 eV<br>3%	-	-	162.1 eV<br>50%	163.4 eV<br>50%	-	+0.4 eV
0.20	2.7	229.2 eV<br>71%	230.0 eV<br>23%	232.3 eV<br>6%		-	162.0 eV<br>55%	163.3 eV<br>45%	-	+0.3 eV
0.35	2.5	229.1 eV<br>77%	230.0 eV<br>17%	232.3 eV<br>6%		-	162.0 eV<br>63%	163.2 eV<br>36%	-	+0.3 eV
0.50	2.2	229.1 eV<br>77%	230.7 eV<br>9%	232.6 eV<br>9%	228.7 eV<br>5%	-	162.0 eV<br>72%	163.4 eV<br>22%	161.0 eV<br>5%	+0.3 eV
0.65	2.1	229.4 eV<br>80%	230.8 eV<br>5%	232.7 eV<br>6%	228.7 eV<br>9%	-	162.3 eV<br>80%	163.5 eV<br>9%	161.4 eV<br>10%	+0.4 eV
0.80	1.9	229.5 eV<br>79%	230.0 eV<br>7%	232.8 eV<br>9%	228.8 eV<br>4%	-	162.4 eV<br>91%	163.0 eV<br>6%	161.5 eV<br>2%	+0.4 eV
0.94	1.3	228.9 eV<br>49%	229.8 eV<br>19%	232.5 eV<br>14%	-	228.6 eV<br>19%	162.3 eV<br>92%	163.8 eV<br>7%	-	+0.4 eV
**300 °C**										
0.20	2.3	229.0 eV<br>78%	229.9 eV<br>16%	232.2 eV<br>2%	228.6 eV<br>3%	-	161.9 eV<br>67%	163.3 eV<br>28%	161.0 eV<br>5%	+0.3 eV
**350 °C**										
0.20	2.1	229.3 eV<br>66%	230.0 eV<br>13%	232.8 eV<br>8%	228.7 eV<br>12%	-	162.2 eV<br>79%	163.5 eV<br>11%	161.3 eV<br>9%	+0.5 eV
0.50	2.2	229.6 eV<br>86%	231.5 eV<br>4%	232.2 eV<br>4%	228.9 eV<br>6%	-	162.4 eV<br>82%	163.7 eV<br>7%	161.6 eV<br>5%	+0.4 eV
0.80	1.9	229.6 eV<br>84%	230.0 eV<br>7%	232.8 eV<br>7%	228.9 eV<br>2%	-	162.4 eV<br>96%	163.7 eV<br>3%	-	+0.6 eV
**450 °C**										
0.20	2.0	229.5 eV<br>77%	230.0 eV<br>7%	232.8 eV<br>7%	228.9 eV<br>8%	-	162.3 eV<br>88%	163.1 eV<br>6%	161.5 eV<br>6%	+0.3 eV
0.80	1.9	229.4 eV<br>86%	230.0 eV<br>3%	232.6 eV<br>6%	228.7 eV<br>5%	-	162.3 eV<br>95%	163.1 eV<br>2%	161.6 eV<br>3%	+0.4 eV

Identification of the low BE Mo component for 94% H_2 dilution samples

For the samples grown using the highest H_2 flow ratio of 0.94, an intense doublet was observed at a lower binding energy compared to the other Mo components, approximately 228.5 eV for Mo $3d_{5/2}$. The BE is lower compared to MoS_2, but somewhat higher compared to typical Mo metal values (227.8±0.5 eV) and closer to values reported for MoN_x (228.4±0.3 eV) and MoC_x (228.3±0.5 eV).[6,7] Therefore, the C and N content of the films was evaluated. C 1s XPS spectra was dominated by atmospheric hydrocarbon contamination (not shown), so an attempt to remove this layer using Ar^+ ion sputtering was made. No clear differences in the carbon concentration were observed for films grown using H_2 flow ratios of 0.20, 0.80, and 0.94, all of which apparently contained ~5 at.% carbon throughout the films. However, this carbon was present at a low binding energy (283–283.5 eV). Thus, we believe the signal to be a result of adventitious surface carbon incorporated into the film as carbidic carbon by Ar^+ ions. Such a process has been observed for several carbide-forming metals of groups 4 to 7.[8–11] Furthermore, no carbon was observed by RBS (detection limit ~7 at-%) in the films grown at 150 °C using H_2 flow ratios of 0.20, 0.65 and 0.80 as discussed in the article.

Analysis of nitrogen by XPS is complicated by overlap of the only XPS relevant orbital, N 1s, with the Mo $3p_{3/2}$ orbital. Furthermore, Mo 3p orbital results in a complicated and asymmetric peak shape. By careful peak fitting and comparison to MoS_xN_y samples where nitrogen was intentionally introduced by adding N_2 into the $H_2S/H_2/Ar$ plasma (these samples are not discussed further in this article), an upper limit to nitrogen content of approximately 5 at.% can be stated for films deposited at any H_2 flow ratio. Thus, it is assumed that the 228.5 eV component mainly corresponds to molybdenum metal with potential minor carbide and/or nitride contributions. Therefore, the films grown using H_2 flow ratio of 0.94 consisted of a mixture of $Mo(C_xN_y)$ and MoS_2.

Variation in Mo 3d and S 2p binding energies

For different deposition conditions (temperature and H_2 flow ratio), the binding energies varied from 288.8 to 229.9 eV for the Mo^{4+} component (Mo $3d_{5/2}$). The shift may have multiple origins, including but not limited to:

1) changes in the chemical environment (which would only shift the particular peak),

2) shift of the Fermi level – if due to doping, a positive shift would correspond to n-type and a negative shift to p-type doping (which would shift all XPS peaks by a similar amount),[12,13]

3) charging effects (which may shift peaks at different BEs by different amounts),

4) changes in the binding energy of adventitious carbon,[14,15] which was used as a BE reference and was fixed to 284.8 eV, depending on the chemical nature and morphology of the MoS_x surface (which would shift all XPS peaks by a similar amount).

While the binding energy shifts are not the main focus of this article, a qualitative treatment suggests that several of these effects and possibly others may be in play. For example, peak shifts to similar direction to Mo 3d were observed for the S 2p peaks and the valence band (Figure S6 and S7), which would be in agreement with origins 2 to 4. The shifts for S 2p were smaller compared to Mo 3d, however, suggesting that also chemical environment changes (or other effects causing non-global peak shifts) may have contributed to the observed shifts.

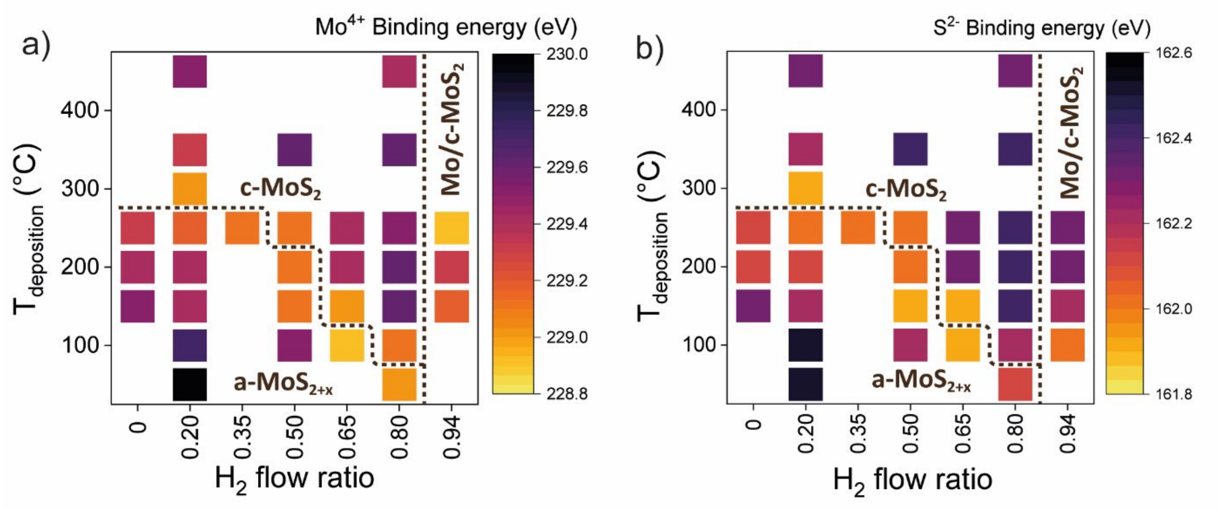

Figure S6. Measured binding energies of a) Mo $3d_{5/2}$ (Mo^{4+}) and b) S $2p_{3/2}$ (**S^{2-}** & S_2^{2-} terminal) as a function of deposition temperature and H_2 flow ratio. The films were deposited using 100–140 ALD cycles.

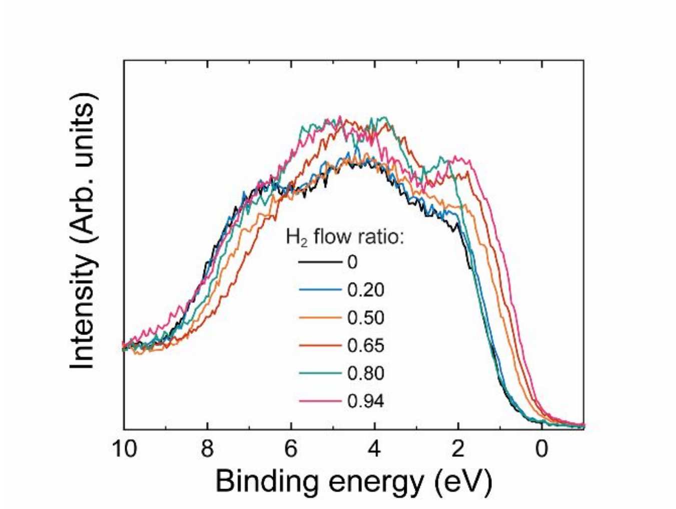

Figure S7. Valence band X-ray photoelectron spectra of films deposited at 150 °C using different H_2 flow ratios.

S6. SEM images

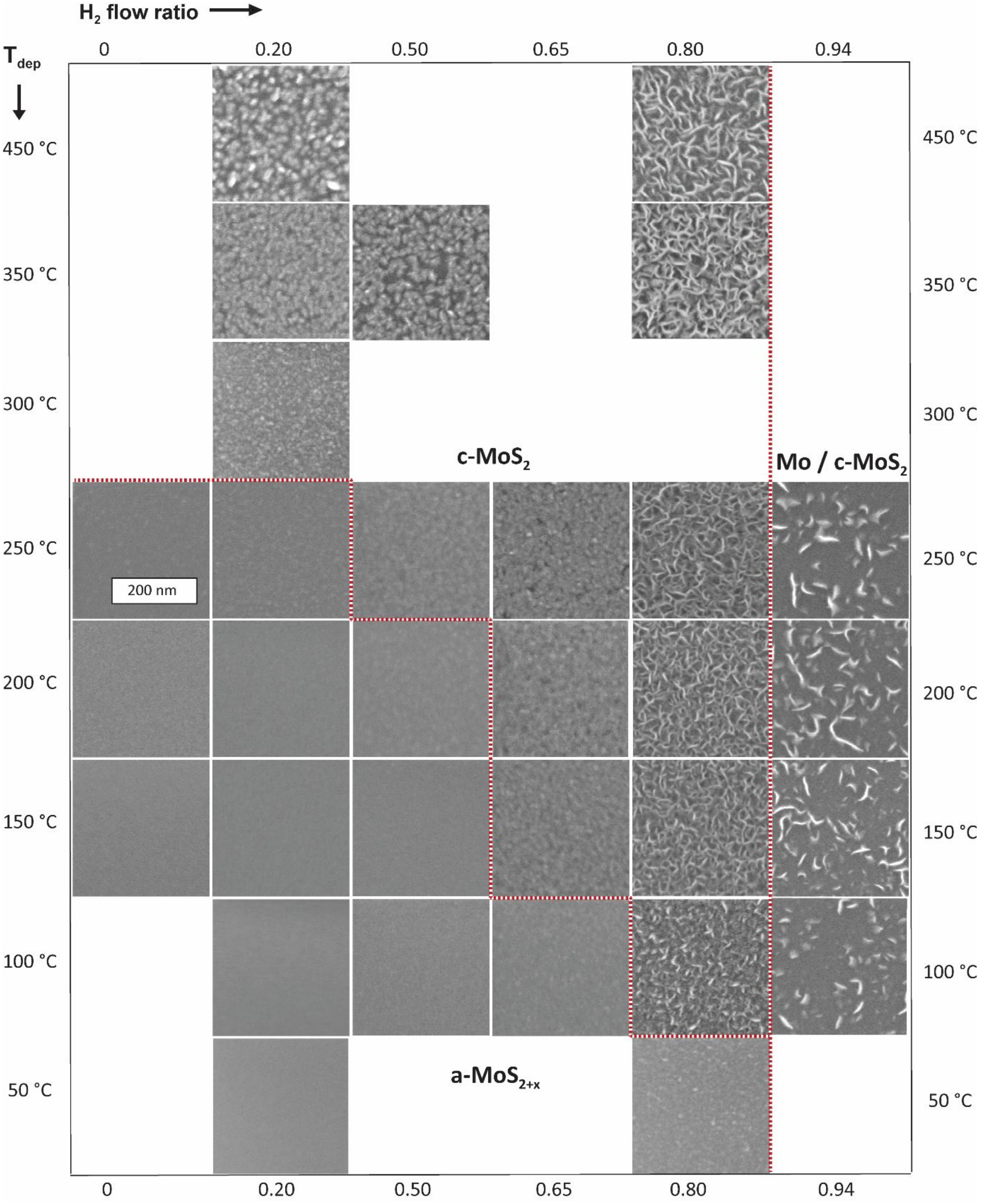

Figure S8. SEM images of MoS_x films deposited in different conditions. All of the images are in the same scale. The films were deposited using 100–140 ALD cycles, resulting in thicknesses ranging from 8 to 20 nm.

S7. Cross-sectional TEM images before and after filtering

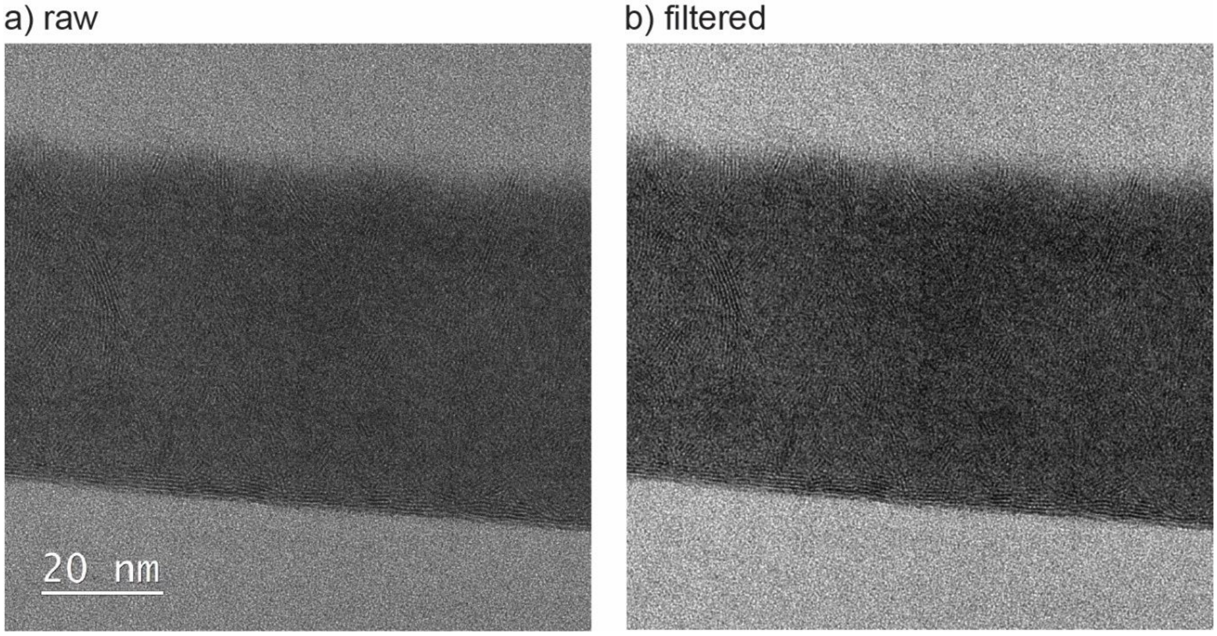

Figure S9. Cross-sectional TEM image of a ~50 nm thick MoS_2 film grown at 150 °C using H_2 flow ratio of 0.65 and 500 ALD cycles a) before and b) after applying a smoothing filter and adjusting brightness and contrast. A cropped, filtered image is shown in Figure 6.

S8. In situ spectroscopic ellipsometry

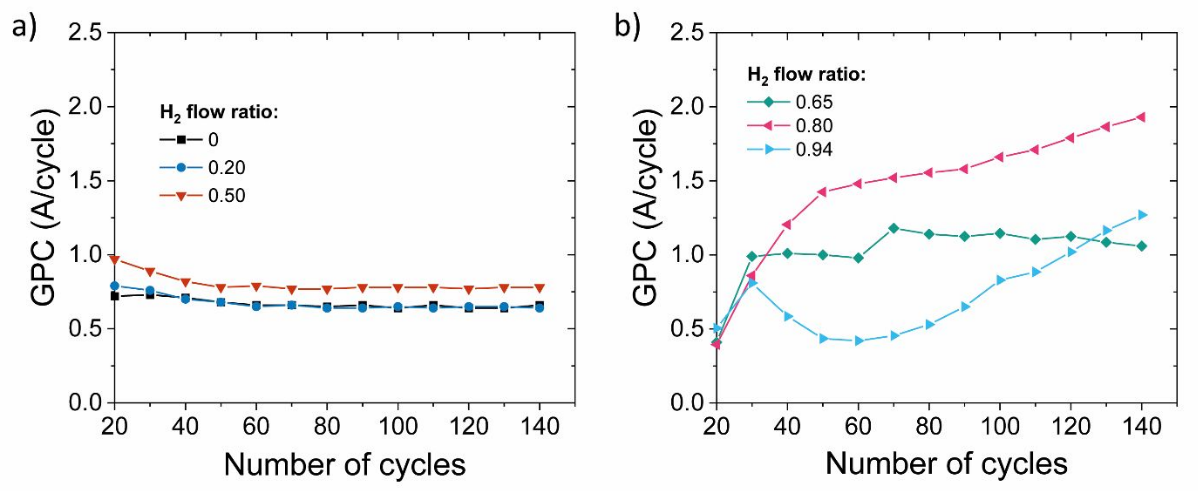

Figure S10. Evolution of GPC as a function of ALD cycles for a) a-MoS_{2+x} and b) c-MoS_2 films deposited at 150 °C using different H_2 flow ratios. The GPCs have been calculated from the data shown in Figure 7a. The first 10 cycles were not included, as the thickness determination at such low thicknesses has a large uncertainty.

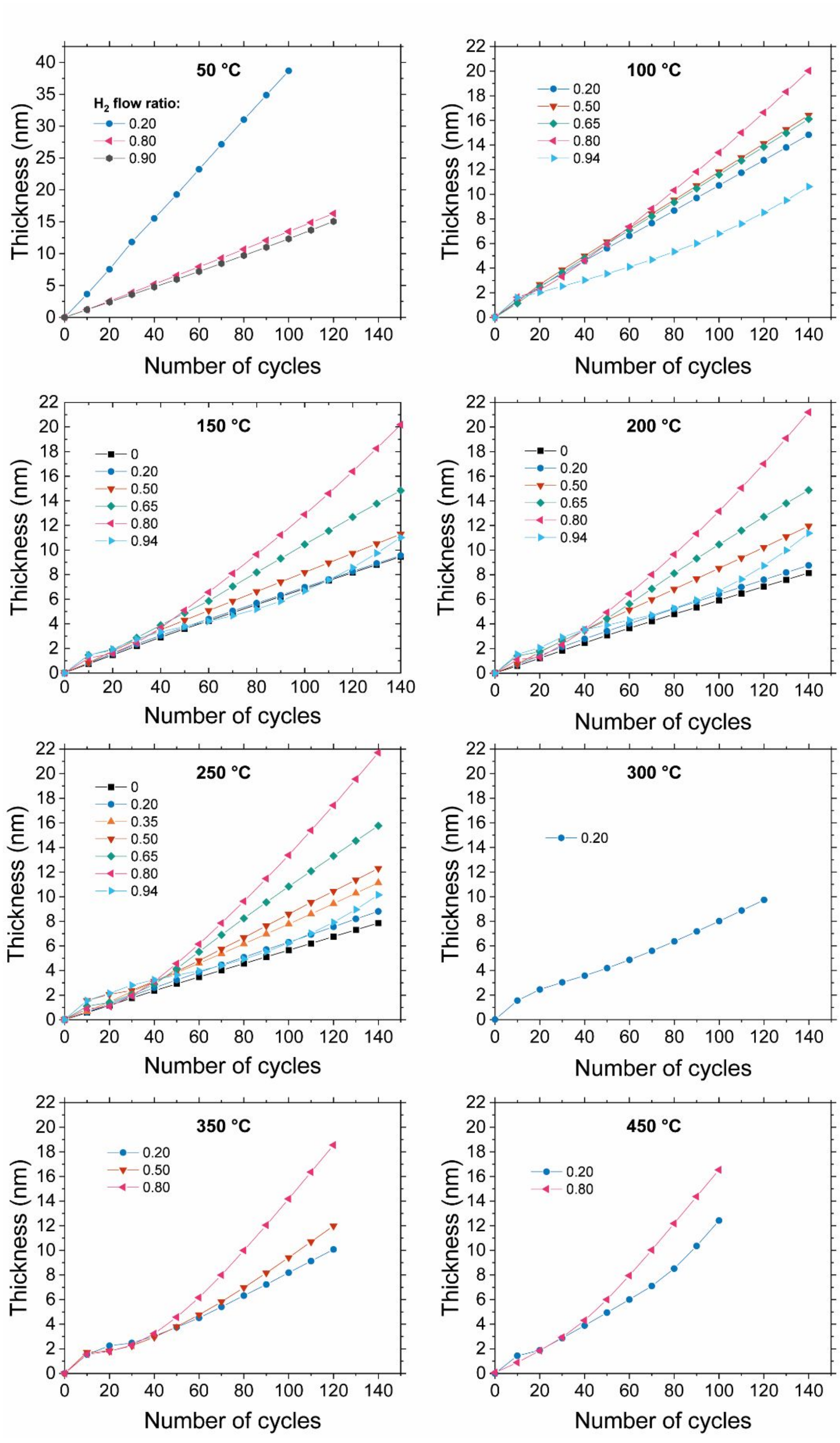

Figure S11. Evolution of film thickness as a function of ALD cycles in different conditions as measured by in-situ SE. Note the different scale in 50 °C depositions compared to the other temperatures.

S9. Tabulated data of samples and their characteristics

Table S2. Summary of film characteristics in tabulated form. Thickness and GPC were determined by in-situ SE, resistivity by combining sheet resistance measured by FPP and SE thickness (detection limit (dl) corresponds to approximately 1000 Ωcm for approximately 10 nm thick films), crystallinity by Raman spectroscopy, and S/Mo ratio by XPS.

T_{dep} (°C)	ALD cyc.	H_2 flow ratio	Thickness (nm)	GPC after 100 cyc (Å/cyc)	Resistivity (Ωcm)	Crystallinity	S/Mo atomic ratio
50	100	0.20	38.5	3.9	>dl	Am	7.3
50	120	0.80	16.3	1.35	0.34	Am	2.0
50	120	0.90	15.1	1.23	0.15	Am	1.8
100	140	0.20	14.8	1.07	>dl	Am	4.6
100	140	0.50	16.4	1.18	>dl	Am	3.6
100	140	0.65	16.1	1.16	2.0	Am	2.2
100	140	0.80	18.5	1.34	0.17	Cryst	1.8
100	140	0.94	10.7	0.68	0.013	Cryst	1.4
150	140	0	9.5	0.69	>dl	Am	3.6
150	140	0.20	9.3	0.67	>dl	Am	3.5
150	140	0.50	11.3	0.82	~1000	Am	3.0
150	140	0.65	14.8	1.04	0.45	Cryst	2.1
150	140	0.80	20.1	1.29	0.35	Cryst	1.9
150	140	0.94	10.9	0.67	0.008	Cryst	1.3
200	140	0	8.2	0.59	>dl	Am	3.1
200	140	0.20	8.4	0.64	>dl	Am	3.0
200	140	0.50	12.0	0.85	6.2	Am	2.5
200	140	0.65	14.9	1.05	0.65	Cryst	2.2
200	140	0.80	21.2	1.31	0.81	Cryst	1.9
200	140	0.94	11.4	0.67	0.0065	Cryst	1.3
250	140	0	7.9	0.57	260	Am	2.8
250	140	0.20	8.8	0.63	65	Am	2.7
250	140	0.35	11.1	0.78	6.5	Am	2.5
250	140	0.50	12.3	0.86	1.5	Cryst	2.2

T_{dep} (°C)	Cyc.	H_2 flow ratio	Thickness (nm)	GPC after 100 cyc (Å/cyc)	Resistivity (Ωcm)	Crystallinity	S/Mo atomic ratio
250	140	0.65	15.8	1.08	1.0	Cryst	2.1
250	140	0.80	21.7	1.34	1.1	Cryst	1.9
250	140	0.94	10.1	0.63	0.0039	Cryst	1.3
300	120	0.20	9.7	0.80	15	Cryst	2.3
350	120	0.20	10.6	0.83	24	Cryst	2.1
350	120	0.50	12.0	1.25	100	Cryst	2.2
350	120	0.80	18.0	1.45	5.2	Cryst	1.9
450	100	0.20	12.5	1.25	220	Cryst	2.0
450	100	0.80	16.5	1.65	0.59	Cryst	1.9

S10. Hall measurements

Hall effect measurements of MoS_x films were performed in van der Pauw geometry using a Lakeshore 8404 HMS instrument as described in the *Experimental section* of the article. Conventional DC Hall effect measurements were unsuccessful due to the low carrier mobility μ of our samples, which is in practice proportional to the measured Hall voltage V_H (this relation can be worked out from simple electromagnetic treatment of Hall measurements presented in e.g. Ref.[16], free electron theory, and assumption of the voltage applied over the sample being the same for each sample). The lower limit of measured V_H in DC Hall measurements is set by other voltages present, including misalignment voltage, thermoelectric voltage, Ettingshausen voltage, Nernst effect voltage, Righi-Leduc voltage, and voltmeter/current meter offsets.[16]

AC Hall measurements enable detection of smaller V_H, as using a phase-sensitive lock-in amplifier allows separation of the AC Hall voltage from other voltages present that are either DC or AC with a different frequency. In an AC Hall measurement, the sensitivity is limited by inductive signals present at -90 degree phase with respect to the applied AC magnetic field.[17] For our samples, the inductive voltage was on the order of 10–50 µV, whereas the extracted V_H were on the order of 10–1000 nV, i.e. one to three orders of magnitude lower. Thus, the measured voltage was dominated by the inductive voltage, which causes the uncertainty in Hall mobility, carrier concentration, and majority carrier type to be large. The mobility and carrier concentration are calculated from the magnitude of the extracted V_H, whereas the majority carrier type is inferred from the phase of V_H.

Care was taken to ensure that the obtained results are as reliable as possible for these challenging samples. This included repeated measurements at different currents to confirm that the measured voltage scaled linearly with current as expected for Hall voltage (in contrast, inductive voltage is independent of the DC excitation current). Once the measurement parameters (DC excitation current and AC lock-in amplifier settings) were optimized, multiple repeated measurements were performed to assess measurement uncertainties. Rather large uncertainties are present in the Hall mobility, carrier concentration, and majority carrier type due to the measured signal mostly consisting of inductive voltage as explained above. The mobilities of the samples are close to the capabilities of the instrument, which the manufacturer indicates to be able to characterize samples with mobilities down to 0.001 $cm^2V^{-1}s^{-1}$.[16]

Further validity checks were performed to assess the magnitude of the extracted carrier concentrations and mobilities. This included measuring other samples using the Hall instrument, which produced results expected for these samples. In particular, Al-doped MoS_2 samples reported by Vandalon et al. exhibiting similar, low V_H below 1 µV have been measured with the same instrument. In this case, the carrier concentration scaled (superlinearly) with Al doping level from estimated 10^{17} cm^{-3} for non-intentionally doped MoS_2 to ~$10^{19}cm^{-3}$ for 3 at-% Al, ~10^{20} cm^{-3} for 6 at-% Al, and ~10^{21} cm^{-3} for 9 at-% Al doping with mobilities simultaneously decreasing from 0.07 to 0.01 $cm^2V^{-1}s^{-1}$.[18]

It is worth nothing that besides the measurement uncertainties, additional uncertainty results from the typical assumptions used in the extraction of carrier mobility and concentration from Hall voltage that may not strictly hold. For example, we assumed the Hall factor r to be unity as no other value for MoS_2 was found, but in reality it is generally larger than unity, which would result in underestimation of mobility and overestimation of carrier concentration.

Thus, we are confident that the order of magnitude of our carrier concentration and mobility values are reasonable, but the low V_H of our samples results in relatively large statistical uncertainty. Systematic errors resulting from difficulty of extracting low V_H and use of $r = 1$ mean that the reported values are likely to represent lower limits for mobility and upper limits for carrier concentration.

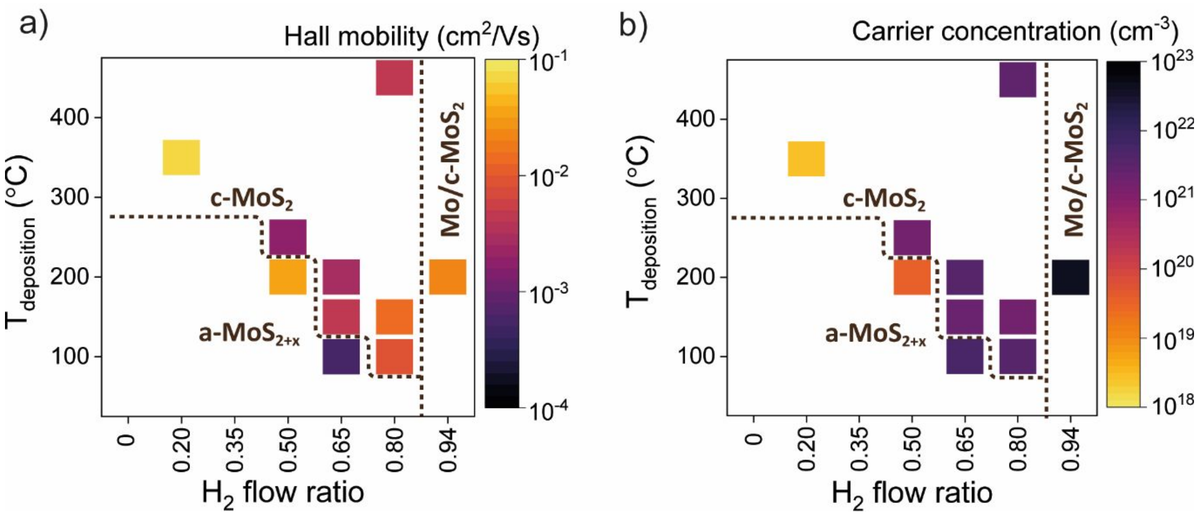

Figure S12. Heatmaps of a) Hall mobility and b) carrier concentration as a function of deposition temperature and H_2 flow ratio.

Table S3. Results of Hall measurements in tabulated form. Additional information on film thickness and Hall measurements is also included. The uncertainties represent standard deviation of usually 9–15 measurements repeated without remounting the sample (5 for 100 °C, 0.65 H_2 flow ratio and 150 °C 0.80 H_2 flow ratio samples, which were confirmed by performing additional measurements with a different DC excitation current). Asterisk (*) in the carrier type indicates that the noted carrier type was found in the majority of the repetitions, but the opposite type also occurred in some repetitions due to low signal-to-noise ratio.

Deposition			Hall measurement		Extracted values		
T_{dep}	H_2 flow ratio	Thick. (nm)	DC current (nA)	V_H (nV)	Hall mobility ($cm^2V^{-1}s^{-1}$)	Carrier concentration (cm^{-3})	Majority carrier type
100	0.65	16.1	250	22±8	$6 \pm 2 \times 10^{-4}$	$6 \pm 4 \times 10^{21}$	p*
100	0.80	18.5	400	60±30	$9 \pm 4 \times 10^{-3}$	$3.4 \pm 1.2 \times 10^{21}$	p
150	0.65	55.6	500	39±16	$4 \pm 2 \times 10^{-3}$	$2.2 \pm 1.1 \times 10^{21}$	p*
150	0.80	20.1	250	83±19	$1.5 \pm 0.3 \times 10^{-2}$	$1.6 \pm 0.5 \times 10^{21}$	p*
200	0.50	12.0	50	330±120	$3.5 \pm 1.3 \times 10^{-2}$	$3.2 \pm 1.3 \times 10^{19}$	p
200	0.65	14.9	1000	190±90	$3.0 \pm 0.9 \times 10^{-3}$	$3.4 \pm 1.3 \times 10^{21}$	p*
200	0.94	11.4	500	9±5	$2.1 \pm 1.3 \times 10^{-2}$	$5 \pm 2 \times 10^{22}$	p*
250	0.50	12.3	125	48±9	$1.6 \pm 0.3 \times 10^{-3}$	$2.0 \pm 0.3 \times 10^{21}$	p
350	0.20	12.0	7	2000±1000	$7 \pm 3 \times 10^{-2}$	$2.7 \pm 1.1 \times 10^{18}$	n*
450	0.80	16.5	100	25±14	$5 \pm 3 \times 10^{-3}$	$3.0 \pm 2.3 \times 10^{20}$	p*

S11. Field effect transistors

To gain insight into the functional properties of MoS_2 grown at low temperatures and elevated H_2 ratios, and to further explore the physical make up of these thin films through electrical characterization, FET devices were constructed. For this, we chose the lowest deposition temperature of 100 °C, in view of compatibility with plastic substrates used in flexible electronics. Thus, an H_2 flow ratio of 0.80 was used to obtain crystalline MoS_2 of approximately 5 nm in thickness. For comparison, reference films were grown at 350 °C using a low H_2 flow ratio of 0.20.

FET devices were fabricated starting with standard metal lift-off process that utilized a reduced photoresist bake temperature of 120 °C followed by fluorine-based ICP dry etching of the channel using resist as mask. Resist mask was then stripped by dipping the samples in acetone. During FET fabrication, in particular photolithographic patterning steps that involve exposure of samples to a photoresist developer, adhesion of both samples to the 90 nm SiO_2/Si (p++) substrate was observed to be poor. A similar situation occurred during the lift-off and dry etching steps, which resulted in poor device yield. Optimization of processing conditions, in particular photoresist baking temperature, allowed some devices to be successfully made and measured.

Comparing output curves (Figure S13a,b, I_{DS} vs. V_{DS}) shows two major differences between the samples. First, the I-V curves of the 100 °C sample exhibited clearly non-linear behavior suggesting Schottky barrier at the contact. In contrast, the IV curves of the 350 °C samples were nearly linear, pointing to the contacts being almost Ohmic in nature. Second, the current at a given voltage is much higher for the 100 °C sample, for example by a factor of 20-50 at V_{DS} = 2 V. For comparison, the four-point resistivity of the 100 °C sample (0.2 Ωcm) prior to FET processing was lower by a factor of 500 compared to the 350 °C sample (100 Ωcm).

Comparison of the transfer curves (Figure S13c,d, I_{DS} vs. V_{GS}) showed p-type behavior of both samples with modest on/off ratios of approximately 3 to 4. The 100 °C sample showed more ambipolar behavior with a more pronounced n-branch compared to the 350 °C sample.

No clear indication of scaling with channel length was observed within the limited number of successfully fabricated devices, which suggests that contact resistance may dominate device behavior. To this end, the MoS_2/metal interfaces were studied as described below. Another point to note is that the aspect ratio of the FET channels (length to thickness) was on the order of 1000–10000:1, which may lead to e.g. surface scattering effects being very significant. Approaches such as capping layers to reduce surface scattering as well as reactivity may be useful to improve device performance. Furthermore, FETs with scaled channel lengths could provide additional insight into the functional electrical properties of these MoS_2 thin films.

Cross-sectional TEM samples were prepared from working FET devices in order to obtain insight into the device behavior. In the channel regions, both samples consisted of crystalline MoS_2 with no obvious damage on the surface or inside the film (Figure S14 a,b). However, the thickness of the MoS_2 layer in the channel region was 1.5 to 2 nm lower compared to that measured by SE. This might suggest partial amorphization of MoS_2 in the channel region (e.g. due to interaction with photoresist and/or oxidation in air), but it could also be due to inaccuracy of the optical model used for SE or formation of an amorphous layer at the SiO_2/MoS_2 interface during film growth as suggested by DFT calculations[19] (such interlayer would be observed by SE but less clearly by TEM).

Underneath the Ni/Au contacts, the thickness and crystallinity of the MoS_2 layer of the 350 °C sample is similar to the channel region. However, a more detailed investigation shows that one to two more MoS_2 monolayers can be counted underneath the contact compared to the channel. This observation

supports the amorphization of the MoS_2 surface in the channel region during FET processing. In addition, an approximately 1 nm thick interface layer that appears lighter in the bright-field TEM image is seen at the Ni/MoS_2 interface for the 350 °C sample. In contrast, for the 100 °C sample, only two to three MoS_2 monolayers are observed underneath the contact, in contrast to four to six in the channel. Correspondingly, the mainly amorphous interlayer between MoS_2 and Ni is much thicker in the 100 °C sample compared to the 350 °C sample. This observation suggests greater reactivity between Ni and MoS_2 for the 100 °C sample resulting in a thicker interlayer and, probably, more pronounced Schottky barrier. The reaction between Ni and metal seems to occur either during Ni deposition or after it at room temperature, as suggested by TEM images showing substantial interlayer formation in a separate 100 °C sample onto which Ni was deposited without any preceding or subsequent patterning or other fabrication steps (images not shown).

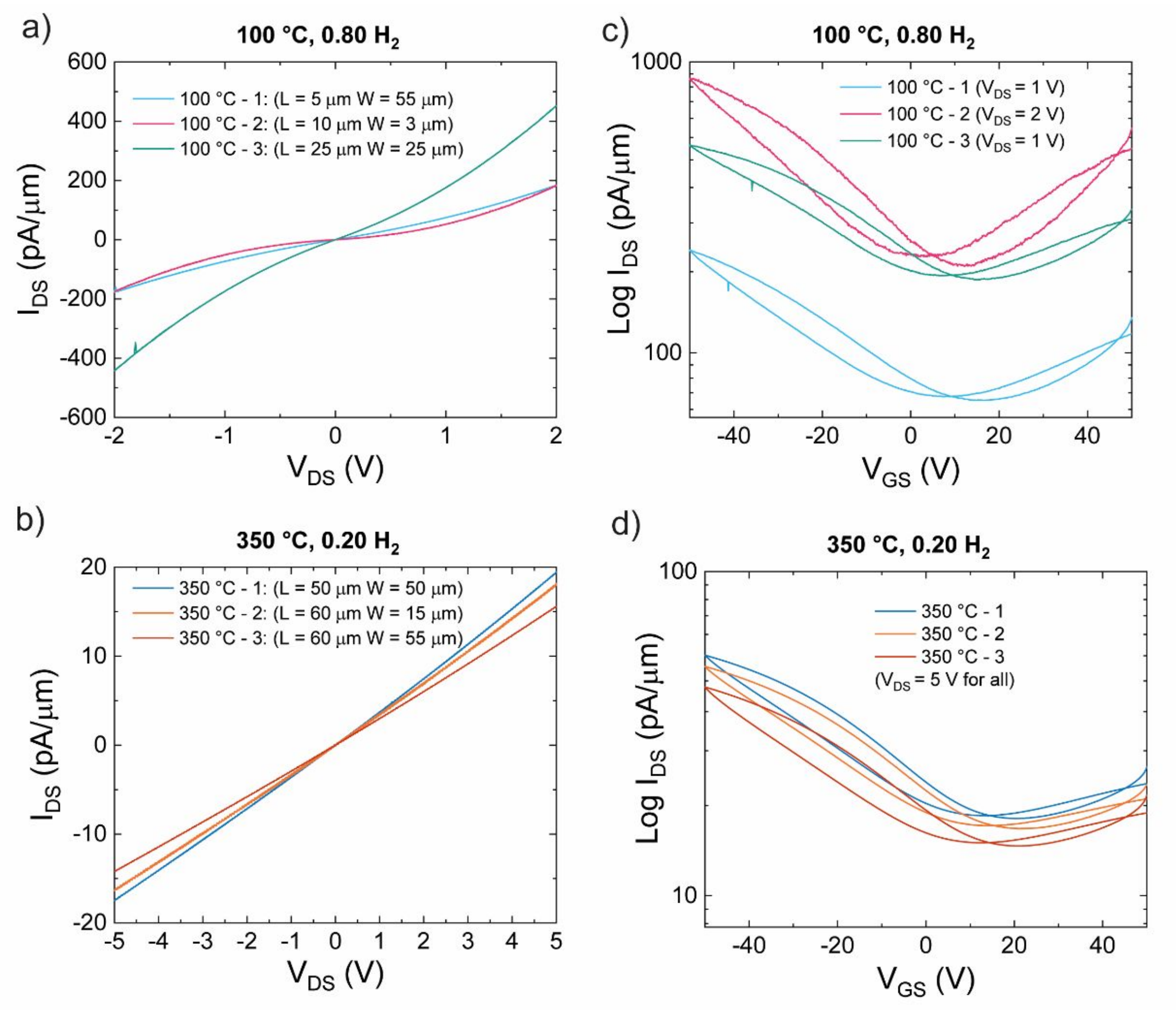

Figure S13. FET characteristics (output and transfer curves) of selected FET devices made using approximately 5 nm MoS_2 films grown at the lowest temperature of 100 °C using H_2 flow ratio of 0.80 and 50 ALD cycles (a,c) and at a reference condition of 350 °C using H_2 flow ratio of 0.20 and 60 ALD cycles (b,d). Three devices for both samples are shown with varying dimensions. D = drain, S = source, G = gate electrode. The currents have been normalized to the channel width.

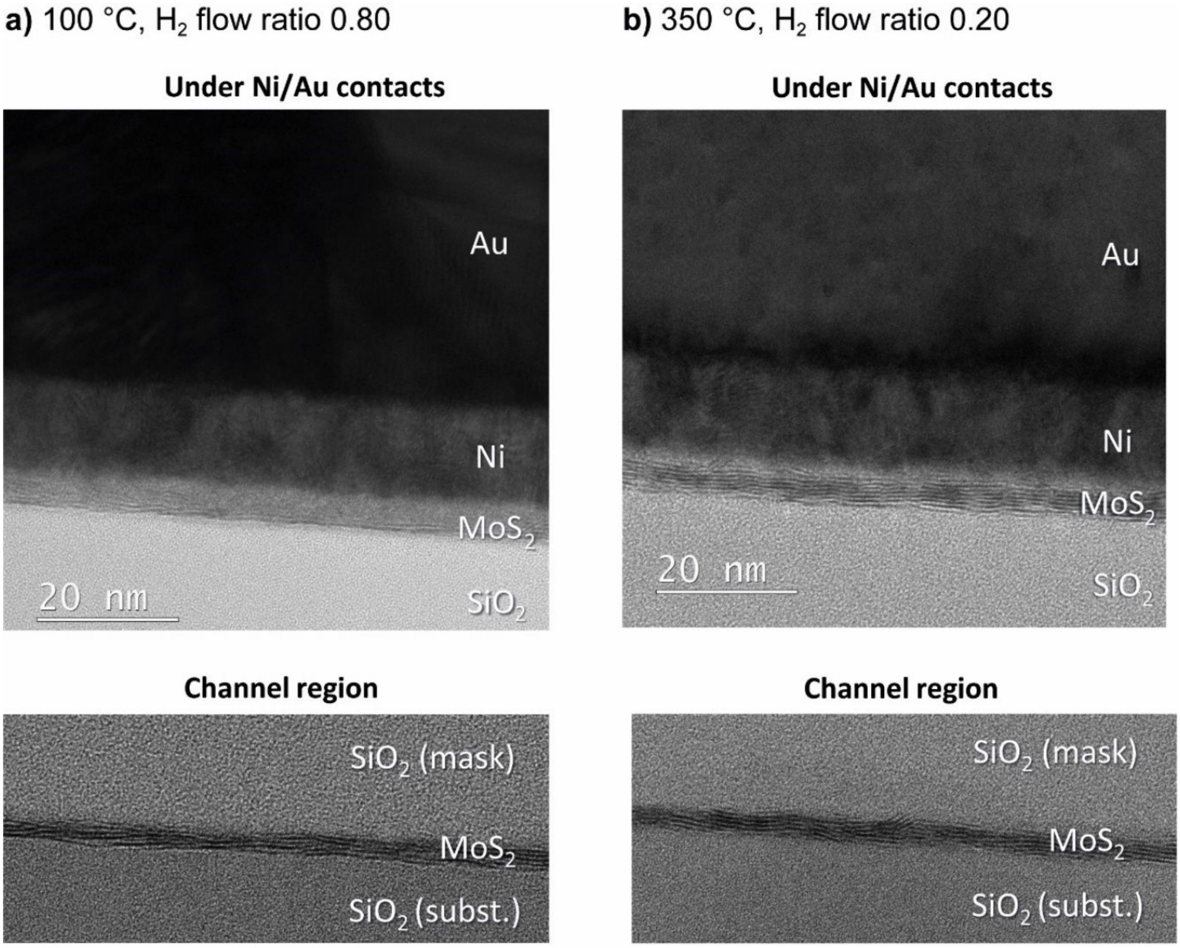

Figure S14. Cross-sectional TEM images of MoS_2 FETs showing the contact and channel regions for films grown at a) 100 °C, H_2 flow ratio 0.80 (50 ALD cycles) and b) 350 °C, H_2 flow ratio 0.20 (60 ALD cycles).

S12. Mild annealing of MoS_2 deposited at low temperatures

A MoS_2 film deposited at 100 °C using an H_2 flow ratio of 0.80 was subjected to annealing under various atmospheres (air, N_2, vacuum, and H_2S) at temperatures ranging from 100 to 300 °C. The motivation of these experiments was two-fold. First, such mild annealing treatments may be encountered in various device fabrication procedures and it is important to know how the material properties change during such treatments. For example, photolithographic processing involves various baking steps in air, whereas annealing in vacuum may be used to improve interface between TMDCs and contact metals. Our FET fabrication procedure included annealing in air at 120 °C for 3 min to bake the photoresist. Second, as hydrogen was found to be incorporated to an increasing degree with increasing H_2 flow ratio, likely leading to heavy doping and high carrier density, we wanted to investigate if the hydrogen could be easily removed from the films.

The annealing treatments were performed for 5 min using a unique 1x1 cm^2 sample for each anneal condition that was cut from a larger piece of a Si wafer after film deposition. Annealing in air and N_2 were done on a hot plate located in a laboratory fume hood and N_2 glove box, respectively. The reported temperatures represent the hot plate surface temperature controlled by the instrument, which was assumed to be close to the sample surface temperature. The annealing experiments in vacuum (~10^{-6} mTorr) and H_2S (200 mTorr) were performed in the ALD reactor used throughout this work. For these experiments, the discrepancy between the set table temperature and actual sample temperature was taken into account using data obtained earlier in our group.

Table S4. Target and setpoint temperatures used for annealing in different annealing atmospheres and instruments used.

T (°C) target	Hot plate setpoint **N_2, air** (°C)	Reactor setpoint 200 mTorr **H_2S** (°C)	Reactor setpoint **vacuum** (10^{-6} mTorr) (°C)
100	100	-	-
150	150	150	165
200	200	240	260
250	250	300	330
300	300	370	420

Four-point-probe measurements showed that resistivity remained unchanged or increased only slightly (reaching no more than ~0.2 Ωcm) from the initial value 0.12–0.15 Ωcm when annealing under N_2, vacuum, or H_2S up to 250 °C (Figure S15). Increasing the annealing temperature to 300 °C resulted in a somewhat larger increase, reaching 0.3 Ωcm (N_2, vacuum) to 0.7 Ωcm (H_2S). In contrast, annealing in air led to a threefold increase of resistivity (to 0.45 Ωcm) after annealing at a temperature as low as 150 °C. Annealing at 200 °C in air increased the resistivity to 5.5 Ωcm and annealing at 250 °C resulted in resistivity exceeding the detection limit of approximately 1000 Ωcm.

Changes in crystallinity upon heating in different atmospheres were examined by Raman spectroscopy (Figure S16). Annealing up to 200 °C resulted in no to very little changes in any of the atmospheres. At 250 °C, annealing in all of the atmospheres besides air resulted in an increase in the intensity of the MoS_2 Raman peaks in reactive (H_2S) and inert (N_2, vacuum) atmospheres. Increasing temperature to 300 °C led to a further intensity increase and also made the differences between the atmospheres more pronounced. As expected, annealing in H_2S led to a higher Raman intensity and presumably better crystallinity compared to inert atmospheres. Annealing in air, in contrast, resulted in practically no change in Raman intensity.

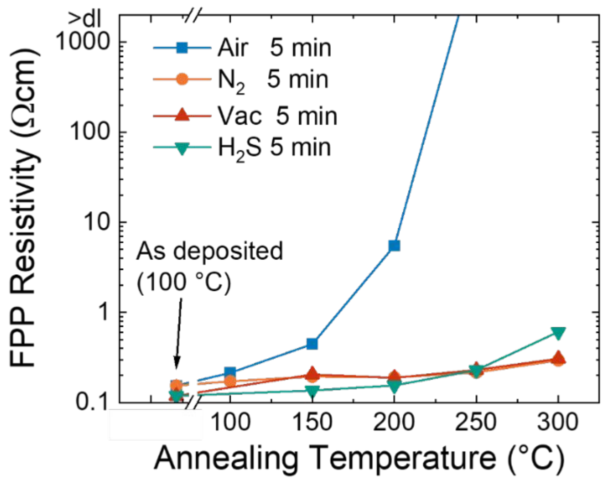

Figure S15. Four-point-probe resistivity of MoS_2 films deposited at 100 °C using H_2 flow ratio of 0.80 (50 ALD cycles) after annealing for 5 min under various atmospheres.

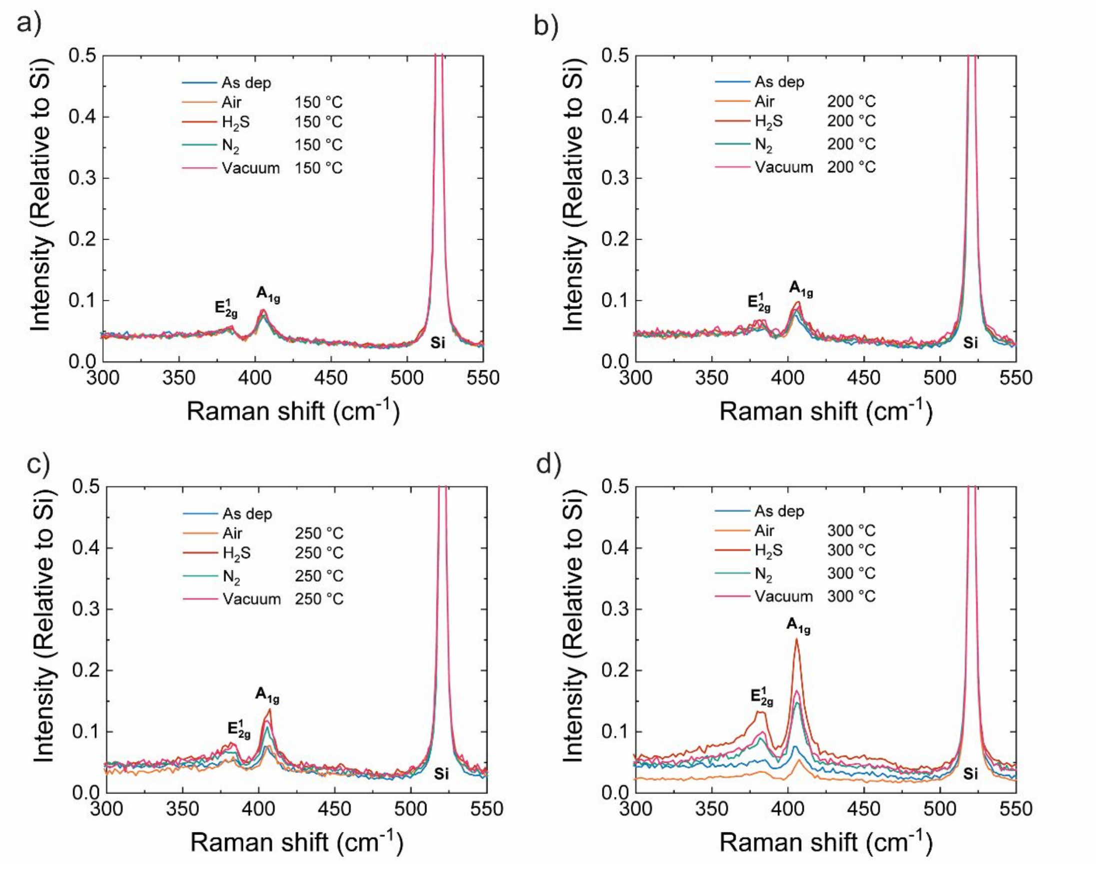

Figure S16. Raman spectra of a MoS_2 film deposited at 100 °C using H_2 flow ratio of 0.80 and 50 ALD cycles after annealing to a) 150 °C, b) 200 °C, c) 250 °C, and d) 300 °C under different atmospheres for 5 min. All of the intensities have been normalized to the Si peak at 520 cm^{-1}.

Selected samples were also subject to XPS measurements to understand chemical changes occurring during annealing (Table S5). Annealing under inert atmospheres at 300 °C increased the area of Mo^{4+} (MoS_x) doublet with a corresponding decrease of Mo^{5+} attributed to oxysulfide and disordered species. H_2S annealing at 300 °C resulted in an even larger increase in the Mo^{4+} content. The changes in S spectra were small (the small increase in S^{6+} content can be attributed to oxidation in air between annealing and XPS measurements). In contrast, annealing in air led to much larger changes. Even at 150 °C, the

amount of oxidized Mo^{6+} species increased notably from 13 to 22% and S/Mo ratio decreased from 1.8 to 1.6. At 200 °C, oxidation was even more prominent with 44% of Mo attributed to Mo^{6+} and S/Mo ratio being as low as 1.2. Interestingly, the change in Mo^{4+} was modest, which may suggest that the most easily oxidized Mo atoms were mostly present as disordered or already partially oxidized Mo^{5+} species. At 250 °C oxidation of MoS_2 continued further, and at this temperature more than half of sulfur was also found to be oxidized. After annealing at 300 °C the film composition was better described as molybdenum sulfate rather than sulfide.

AFM showed that annealing in air led to a modest decrease of roughness from 0.7 nm for the as-deposited sample to 0.6 nm after annealing at 150 °C and 0.5 nm at 200 °C (images not shown).

The changes in MoS_2 film deposited at 100 °C upon annealing up to 300 °C can be summarized as follows. Annealing in air at temperatures as low as 150 °C for 5 min resulted in partial oxidation and increase of resistivity. Annealing at higher temperatures – and presumably longer annealing duration even at 150 °C – cause further oxidation to occur. Thus, care should be taken when applying device processing steps that involve heating in air for these low-temperature MoS_2 films.

Annealing under inert atmospheres up to 300 °C resulted in a modest improvement in crystallinity and modest increase of resistivity. Under an H_2S atmosphere, a somewhat higher change in both was observed. However, in terms of film quality, deposition of films at a higher temperature is a more favorable option compared to annealing of films deposited at low temperatures. The limited increase in resistivity upon annealing further suggests that hydrogen, which is presumed to be responsible for the low resistivity of the films, is not removed to a considerable degree at temperatures up to 300 °C. Additional experiments on H_2S annealing at 350 °C for 1 h showed a more notable increase in resistivity, however both the resistivity and crystallinity remained lower compared to MoS_2 deposition at this temperature.

Table S5. Results of XPS peak fitting for a sample deposited at 100 °C using H_2 flow ratio of 0.80 and 50 ALD cycles as deposited and after annealing at various atmospheres for 5 min. The table contains S/Mo ratios and relative amounts of different Mo and S components. Note that electron-rich $Mo^{(4-\delta)+}$ and S^{2-} species were not included in this fitting procedure.

			Mo $3d_{5/2}$			S $2p_{3/2}$			
Atm.	T (°C)	S/Mo	Mo^{4+} (MoS_x)	Mo^{5+} (MoO_xS_y)	Mo^{6+} (MoO_3)	S^{2-} & S_2^{2-} (terminal)	S_2^{2-} (bridg.) & S^{2-} (apical)	S^0	S^{6+} (sulfate)
Asdep	100	1.8	63%	24%	13%	95%	5%	-	-
Air	100	1.7	58%	26%	16%	92%	8%	-	-
	150	1.6	56%	22%	22%	92%	8%	-	-
	200	1.2	48%	8%	44%	89%	11%	-	-
	250	1.3	29%	55%	66%	46%	-	14%	40%
	300	1.0	2%	15%	83%	24%	-	15%	61%
N_2	300	1.8	80%	9%	11%	92%	-	-	8%
H_2S	300	2.1	88%	6%	6%	90%	5%	-	4%
Vac.	300	2.0	82%	7%	11%	86%	7%	-	8%

S13. Other TMDCs: TiS_x and WS_x

Table S6. Summary of properties of WS_x films deposited at 150 °C using different H_2 flow ratios.

T_{dep} (°C)	H_2 flow ratio	Thickness (nm)	Resistivity (Ωcm)	Crystallinity	S/W ratio
150	0	7.5	>1000	Amorphous	4.0
150	0.80	9.4	4.1	Crystalline	2.1

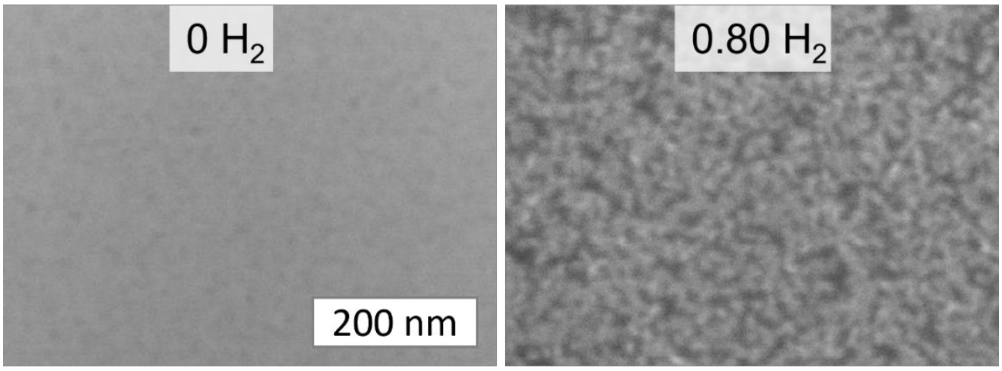

Figure S17. SEM images of WS_x films deposited at 150 °C using different H_2 flow ratios.

Table S7. Summary of properties of TiS_x films deposited at 100 °C using different H_2 flow ratios as well as with thermal ALD process.

T_{dep} (°C)	H_2 flow ratio	Thickness (nm)	Resistivity (Ωcm)	Crystallinity	S/Ti ratio
100	0	16.4	>1000	Amorphous	3.1
100	0.50	10.3	0.028	Crystalline	1.5
100	Thermal ALD	14.6	0.0022	Crystalline	1.2

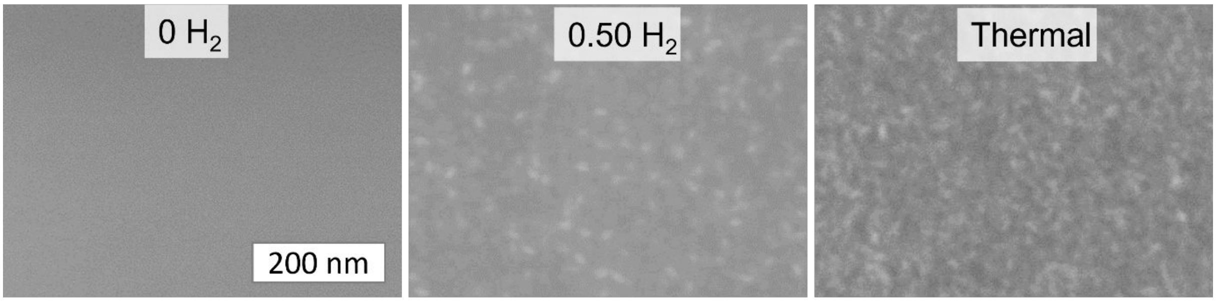

Figure S18. SEM images of TiS_x films deposited at 100 °C using different H_2 flow ratios as well as with thermal ALD process.

S14. Insight into the roles of hydrogen (and process conditions)

A summary of the different factors affecting sulfur incorporation including H_2 flow ratio, H_2S flow rate, deposition temperature, and crystallinity of film surface is presented in Figure S19. A more detailed description is given in the discussion and results presented below.

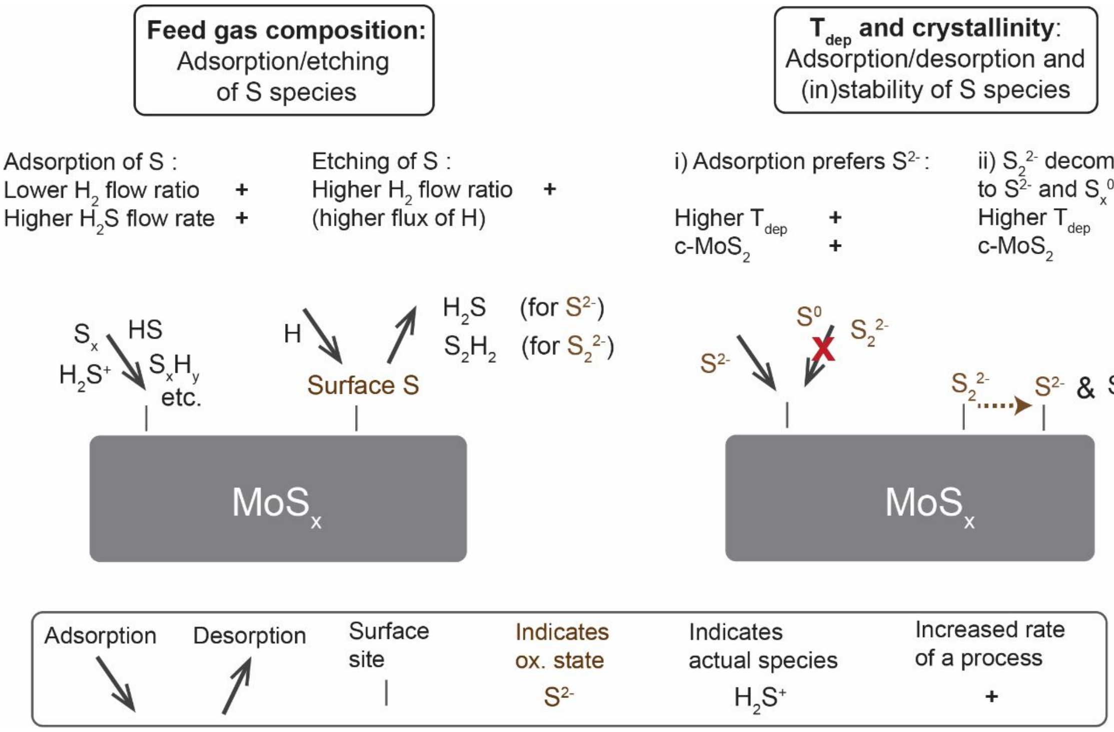

Figure S19. Schematic of different factors and process parameters affecting incorporation of sulfur during low-temperature PEALD of MoS_x and other TMDCs, including feed gas composition (H_2 flow ratio and H_2S flow rate), deposition temperature, and crystallinity/coordination environment of surface. Additionally, the absolute and relative amounts of different S species produced are likely changed as a function of feed gas composition, which can affect S incorporation.

Plasma species

Optical emission spectroscopy (OES) can be used to probe species produced in the plasma discharge.[20] The measurements discussed here were obtained using an Ocean Optics USB4000 spectrometer coupled to the reactor with an optical fiber. The end of the fiber was placed onto a window on top of the ICP tube of the Oxford Instruments FlexAL reactor. As the reaction chamber is located underneath the ICP source, in this geometry the fiber has a line-of-sight into the chamber through the plasma source. Due to the intense emission of plasma, the measurements mostly probe the excited species formed in the plasma discharge.

Figure S20 shows OES spectra recorded at different H_2 flow ratios using plasma conditions comparable to the ALD experiments (total flow rate 50 sccm, of which 40 sccm Ar, at a pressure of 6 mTorr and ICP power of 100 W). For comparison, an H_2/Ar plasma without any H_2S was also investigated (H_2 flow ratio of 1.0). In this case, several peaks attributed to excited atomic argon and both atomic (Hβ and Hγ from Balmer series) and molecular hydrogen (Fulcher band) can be observed between 350 and 650 nm.[21]

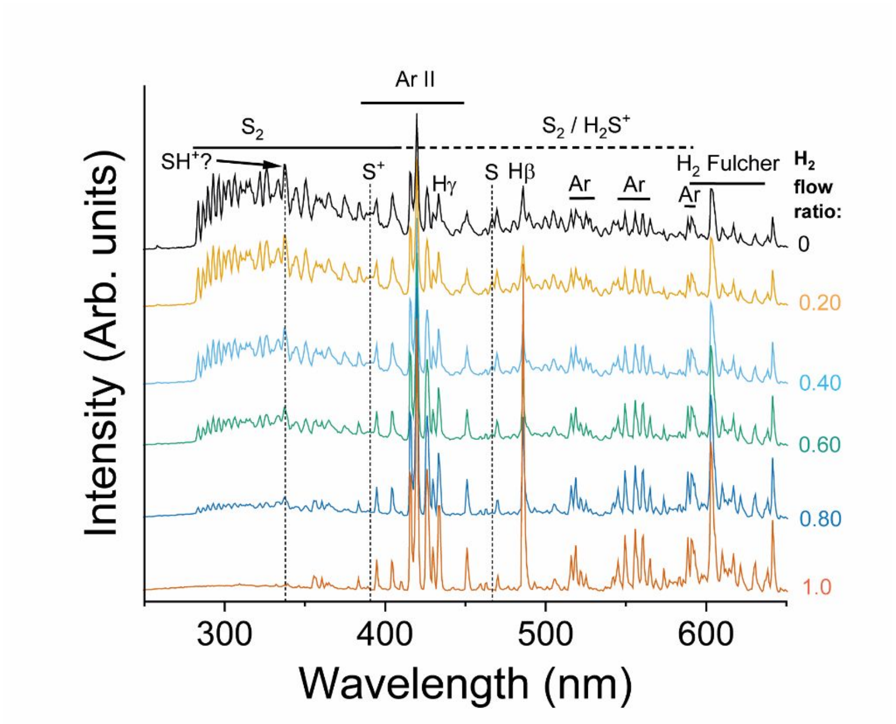

Figure S20. OES spectra of $H_2S/H_2/Ar$ plasma with different H_2 flow ratios.

In plasmas containing H_2S, many peaks attributed to sulfur-containing species were observed between 280 and 600 nm. Although understanding of $H_2S/H_2/Ar$ plasmas is limited, it is known that in a plasma H_2S can dissociate and further react into several sulfur-containing species including (but likely not limited to) H_2S^+, SH, SH^+, S, S^+, and S_2.

Molecular sulfur in the form of S_2 is known to give rise to a plethora of peaks between 280 and 600 nm.[22,23] The reported 260 nm emission of S_2 (or its second order peak at 520 nm) was not observed.[24] Radical ion H_2S^{+*} has been shown to result in many emission peaks between 400 and 500 nm.[24,25] Furthermore, the observed peak at 469.5 nm has been attributed to atomic sulfur[22,23], and the peak at 393.3 nm to S^+ ions.[22] The peak at 336 nm has also been attributed to SH^+ ions, although it also overlaps with S_2 emission.[26]

Potential reaction equations described in the literature are discussed in the following, although it should be noted that these have been reported under different plasma conditions and no kinetic data is available, making them only illustrative of potential reactions:

H_2S^+ may be formed by direct ionization of H_2S:[27]

$H_2S + e^- \rightarrow H_2S^+ + 2e^-$

This can be followed by dissociative neutralization:[27]

$H_2S^+ + e^- \rightarrow HS + H$

Alternately, H_2S can be directly dissociated following reaction: [27]

$H_2S + e^- \rightarrow HS + H + e^-$

HS radicals formed by e.g. above reactions can lead to formation of S:[27]

$H + SH \rightarrow H_2 + S$

Sulfur atoms can then combine to form S_2

$S + S \rightarrow S_2$

Dissociation of S_2 can also occur:[28]

$S_2 + e^- \rightarrow S^* + S + e^-$

Charge transfer from metastable (m) ionized Ar to sulfur species has also been mentioned as an important mechanism in H_2S/Ar plasmas:[23]

$Ar^{+m} + S \rightarrow Ar + S^+$

$Ar^m + H_2S \rightarrow H + HS + Ar$

Observing the intensity of peaks originating from sulfur species shows a linear increase with decreasing H_2 flow ratio, as exemplified for the 336 nm peak in Figure S21a. This is in line with the corresponding increase of H_2S flow rate (see *Experimental* section of the article). Thus, although the S species present could not be identified with certainty, it is clear that their amount increased with decreasing H_2 flow ratio, in line with flow rate changes of H_2 and H_2S.

The situation for atomic H, assumed to be the main reactive H species for ALD, seems less straightforward (Figure S21b). Although decreasing H_2 dilution decreased intensity of the peaks (here, Hα at 656.3 nm, which is just outside the range shown in Figure S20). The strong decrease in the 656.3 nm emission intensity when only a little H_2S was added (H_2 flow ratio of 0.80) may be due to optical absorption or changes in plasma chemistry. The detection of H even with H_2 flow ratio of 0, on the other hand, can be understood by production of H via dissociation of H_2S as discussed above. These two effects may explain the non-linear change in Hα emission intensity between H_2 flow ratios of 0 and 0.80. Furthermore, it is worth noting that in general the intensity of OES peaks cannot be easily translated into the density of the plasma species due to effects such as existence of several excitation channels as discussed in Ref.[20] Nevertheless, as expected, increase of H_2 flow ratio seems to decrease amount of S species and increase amount of H generated in plasma. Thus, changes in the ratio of S to H species are pronounced.

This suggests that increasing the H_2 flow ratio not only increases the absolute amount of H reaching the substrate, but also increases the ratio of H to S species. As discussed in the following, we believe that both the absolute and relative amounts of H and S species are important in controlling MoS_x stoichiometry and crystallinity.

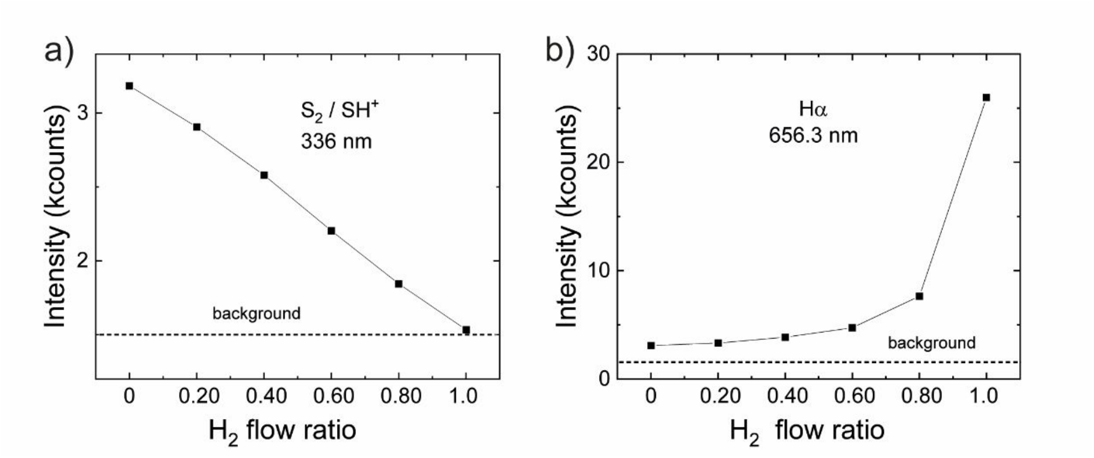

Figure S21. Emission intensity at a) 336 nm and b) 656.3 nm obtained from OES spectra measured at different H_2 flow ratios.

Effect of total and H_2S partial pressures

The experiments described in this article were designed such that the total flow rate into the plasma tube was kept constant at 50 sccm, of which 40 sccm was Ar and the combined flow rate of H_2 and H_2S was 10 sccm. Therefore, increasing the H_2 flow ratio resulted in a decrease of H_2S flow rate (see the *Experimental* section for more details). This raises the question if simply decreasing H_2S flow rate without any additional H_2 flow would have a similar effect.

At temperatures where relatively little excess S is incorporated, H_2S flow rate indeed has a clear effect on stoichiometry and crystallinity. This is exemplified in Figure S22a, showing that crystalline films could be deposited at 250 °C by decreasing the H_2S flow rate from the standard 10 sccm to 2 sccm, without any additional hydrogen. In contrast, at lower temperatures, such as 150 °C, addition of H_2 was necessary to deposit crystalline films, at least within the limits of H_2S flow rates that we can achieve in our setup (Figure S22b). Finally, H_2S flow rate also has an effect on S incorporation and crystallinity when higher H_2 flow ratios are used. For example, at 150 °C and H_2 flow ratio of 0.65 – the lowest H_2 flow ratio used to deposit crystalline films at this temperature – crystallinity could be improved by decreasing the H_2S flow rate from the standard 3.5 to 2 sccm, while an increase of H_2S flow rate to 7 sccm led to deposition of amorphous, sulfur-rich films (Figure S22c). Thus, a decrease of H_2S flow rate acts to the same direction to an increase of H_2 flow ratio and an increase of H_2S flow rate is similar (although less effective) to a decrease of H_2 flow ratio. Furthermore, an increase of H_2S flow rate can be compensated for by an increase of H_2 flow ratio.

Thus, at the same time that have identified H_2S flow rate as an important parameter – which will plan to examine in more detail in the future – we have confirmed that hydrogen plays an active role and is in fact necessary for deposition of MoS_2 at low temperatures in our ALD reactor.

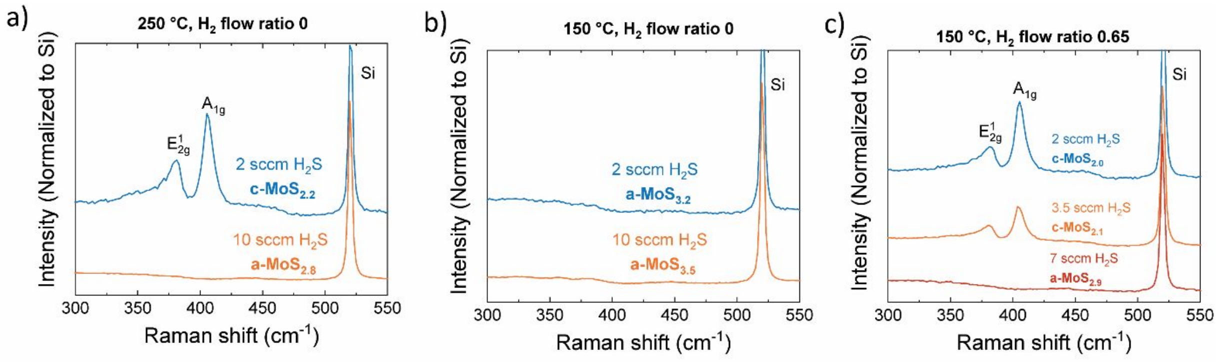

Figure S22. Raman spectra of MoS_x films grown at with varying H_2S flows but fixed H_2 flow ratios: a) 250 °C, H_2 flow ratio 0, b) 150 °C, H_2 flow ratio 0, and c) 150 °C, H_2 flow ratio 0.65. Stoichiometries determined by XPS are also shown. The Raman data has been offset vertically for clarity.

Surface reactions

After identifying the effect of H_2 and H_2S flow rates on plasma species generated and sulfur incorporated into the films, we discuss some surface reaction aspects connecting the gas phase species with the resulting films. The surface reactions taking place in our MoS_x process are not known. Even in the literature, there is limited knowledge on the surface reactions occurring during PEALD of sulfides. Relevant insights can be obtained from the work of Kuhs and coworkers, who have investigated reaction mechanisms during PEALD of Al_2S_3, Ga_2S_3, and ZnS using metal alkyl precursors ($AlMe_3$, $GaMe_3$, and $ZnMe_2$) and H_2S/Ar plasma.[29–31] The reaction mechanisms were mainly observed to be of ligand exchange type, where alkane (ethane or methane, a protonated form of precursor ligand) is released as a byproduct during both precursor pulses. Ligand exchange during metal precursor pulse suggests that -SH groups are present on the growing sulfide surface. Ligand exchange during H_2S plasma, on the other hand, suggests that species containing both hydrogen and sulfur (e.g. SH, H_2S) take part in surface reactions. Besides, a lower amount of CS_2 suggesting "combustion" type reactions was observed. In these reactions, besides the species active in ligand exchange, species containing only sulfur such as S and S_2 may be active.

The imido and amido ligands of our Mo precursor are quite different from the alkyl ligands studied by Kuhs et al. However, we propose that similar ligand exchange reactions may take place and combustion type reactions producing NS_x species may also be possible. Our situation is further complicated by the oxidation state of Mo, which is +6 in the precursor but +4 in MoS_2. Reduction of molybdenum may be achieved by hydrogen radicals – there is an ALD process for WS_2 using WF_6, H2 plasma, and H_2S – where this unambiguously takes place.[32] Due to the abundance of H created from H_2S dissociation, H radicals may act as reducing agent also in case of MoS_2. From other reported ALD processes, it can also be inferred that H_2S, and likely also SH radicals, can act as reducing agents in ALD processes.[33]

Throughout the article, we have shown that S/Mo stoichiometry can be controlled by deposition temperature, H_2 flow ratio, and H_2S flow rate. Especially for the highest S/Mo ratios, questions arise on how such amounts of S are incorporated, and whether such reactions are self-limiting. The latter was shown to be the case by increasing the length of the plasma step at H_2 flow ratio of 0.20 at 150 °C from 10 to 20 and 40 s, which did not result in an increase of S/Mo ratio (approximately 3.5). Therefore, the resulting S/Mo ratio is controlled by the temperature and plasma composition in self-limiting manner.

A potential pathway to explain the S rich stoichiometry may be SH radicals or S_xH_y species formed in H_2S plasma. These contain sulfur at an oxidation state of either -1 (end of S-S chains) or 0 (in the middle of a chain). For example, SH or S_2H_2 species could result in a single sulfur atom deposited per ligand

removed in contrast to H_2S removing 2 ligands and depositing only a single S atom, and thus result in sulfur rich stoichiometry. Furthermore, the oxidation state of -1 for S would match the oxidation state in which excess S is found to be present in our films. While there are likely abundant S_2 (and other S_n) species formed in the plasma, these would need to be reduced to reach the oxidation states of -1 or -2 that are observed for S in the films. Thus, while the precise mechanism leading to excess S incorporation to our MoS_x films is unclear, there are several possible pathways.

Finally, we discuss how the excess S can be removed or its incorporation prevented. Increasing temperature is one option, as shown also by us – no additional H_2 is needed to deposit stoichiometric MoS_2 at 300 °C and above. This can be understood by decomposition of MoS_3 to MoS_2, where a fraction of S_2^{2-} species may be oxidized to form elemental S which is then vaporized, reducing the remaining S_2^{2-} to S^{2-} in the process. As increasing temperature is not an option when using thermally sensitive substrates, for example, in this work we turned to modifying plasma condition to remove excess S and/or to prevent its incorporation. Although distinguishing these two experimentally is very difficult, we will show proof for the ability of H to remove excess S in the following.

Ability of reactive H species to remove excess S

The experiments described above have proven the importance of H_2 in the plasma feed gas in obtaining crystalline MoS_2 films at low temperatures. To gain direct evidence on the ability of hydrogen to control sulfur incorporation, we added a separate hydrogen gas or plasma step after the $H_2S/H_2/Ar$ plasma step. The resulting cycle can be called an "ABC" cycle, where A is the $Mo(N^tBu)_2(NMe_2)_2$ pulse, B is $H_2S/H_2/Ar$ plasma step using H_2 flow ratio of 0.20, and C is H_2 gas or H_2 plasma step. At 150 °C and H_2 flow ratio of 0.20, the AB process produces a-$MoS_{3.4}$ films (Table S8). Addition of an H_2 gas step (ABC_g) had no effect on film composition, showing that H_2 gas is inefficient in removing sulfur and that the films do not spontaneously lose the excess sulfur at such low temperatures. Using H_2 plasma step (ABC_p) instead resulted in crystalline $MoS_{1.9}$ films, showing that reactive hydrogen species (radicals and/or ions) produced in plasma are capable of removing excess sulfur, resulting in crystalline and stoichiometric MoS_2 films.

Table S8. Evidence that species formed in H_2 plasma are able to remove excess sulfur from the films, while H_2 gas is unable to do so. In this case, H_2 flow ratio of 0.20 was used for the B step, after which an additional H_2 gas or H_2 plasma step was added. All of the depositions were done at 150 °C using 140 cycles.

Process	Plasma step (B)	Additional step (C)	Resistivity (Ωcm, FPP)	Crystallinity (Raman)	S/Mo (XPS)
AB	$H_2S/H_2/Ar$ H_2 flow ratio 0.20	-	>1000	Amorphous	3.4
ABC_g	$H_2S/H_2/Ar$ H_2 flow ratio 0.20	H_2 gas	>1000	Amorphous	3.6
ABC_p	$H_2S/H_2/Ar$ H_2 flow ratio 0.20	H_2 plasma	5.7	Crystalline	1.9

References

(1) Sharma, A.; Verheijen, M. A.; Wu, L.; Karwal, S.; Vandalon, V.; Knoops, H. C. M.; Sundaram, R. S.; Hofmann, J. P.; Kessels, W. M. M.; Bol, A. A. Low-Temperature Plasma-Enhanced Atomic Layer Deposition of 2-D MoS_2: Large Area, Thickness Control and Tuneable Morphology. *Nanoscale* **2018**, *10*, 8615–8627.

(2) Vos, M. F. J.; Macco, B.; Thissen, N. F. W.; Bol, A. A.; Kessels, W. M. M. Atomic Layer Deposition of Molybdenum Oxide from $(N^tBu)_2(NMe_2)$2Mo and O_2 Plasma. *J. Vac. Sci. Technol. A.* **2016**, *34*, 01A103.

(3) Afanasiev, P.; Bezverkhy, I. Synthesis of MoS_x (5 < x < 6) Amorphous Sulfides and Their Use for Preparation of MoS_2 Monodispersed Microspheres. *Chem. Mater.* **2002**, *14*, 2826–2830.

(4) Zhang, W.; Zhou, T.; Zheng, J.; Hong, J.; Pan, Y.; Xu, R. Water-Soluble MoS_3 Nanoparticles for Photocatalytic H_2 Evolution. *ChemSusChem* **2015**, *8*, 1464–1471.

(5) Huang, Z.; Wang, C.; Pan, L.; Tian, F.; Zhanbg, X.; Zhang, C. Enhanced Photoelectrochemical Hydrogen Production Using Silicon Nanowires@MoS_3. *Nano Energy* **2013**, *2*, 1337–1346.

(6) Greczynski, G.; Primetzhofer, D.; Hultman, L. Reference Binding Energies of Transition Metal Carbides by Core-Level x-Ray Photoelectron Spectroscopy Free from Ar^+ Etching Artefacts. *Appl. Surf. Sci.* **2018**, *436*, 102–110.

(7) NIST X-ray Photoelectron Spectroscopy Database, Version 4.1 (National Institute of Standards and Technology, Gaithersburg, MD, 2012); http://srdata.nist.gov/xps/ (accessed 24 September 2021).

(8) Holm, R.; Storp, S. ESCA Studies on Changes in Surface Composition under Ion Bombardment. *Appl. Phys.* **1977**, *12*, 101–112.

(9) Luthin, J.; Plank, H.; Roth, J.; Linsmeier, C. Ion Beam-Induced Carbide Formation at the the Titanium-Carbon Interface. *Nucl. Instrum. Methods Phys. Res., Sect. B* **2001**, *182*, 218–226.

(10) Engelhard, M.; Herman, J.; Wallace, R.; Baer, D. As-Received, Ozone Cleaned and Ar^+ Sputtered Surfaces of Hafnium Oxide Grown by Atomic Layer Deposition and Studied by XPS. *Surf. Sci. Spectra* **2011**, *18*, 46–57.

(11) Selvam, P.; Viswanathan, B.; Srinivasan, V. Ion-Induced Carbide Formation of TiFe: Evidence from XPS and AES Studies. *J. Less-Common Met.* **1990**, *161*, 77–85.

(12) Sezen, H.; Suzer, S. Enhancement of Dopant Dependent x-Ray Photoelectron Spectroscopy Peak Shifts of Si by Surface Photovoltage. *J. Chem. Phys.* **2011**, *135*, 141102.

(13) Sezen, H.; Suzer, S. XPS for Chemical- and Charge-Sensitive Analyses. *Thin Solid Films* **2013**, *534*, 1–11.

(14) ASTM Standard E1523-15: Standard Guide to Charge Control and Charge Referencing Techniques in X-Ray Photoelectron Spectroscopy (ASTM International, West Conshohocken, PA, 2015).

(15) Baer, D. R.; Artyushkova, K.; Cohen, H.; Easton, C. D.; Engelhard, M.; Gengenbach, T. R.; Greczynski, G.; Mack, P.; Morgan, D. J.; Roberts, A. XPS Guide: Charge Neutralization and Binding Energy Referencing for Insulating Samples. *J. Vac. Sci. Technol. A* **2020**, *38*, 031204.

(16) Lindemuth, J. *Hall Effect Measurement Handbook*; Lake Shore Cryotronics, Westerville, OH.

(17) Lindemuth, J.; Mizuta, S.-I. Hall Measurements on Low-Mobility Materials and High Resistivity Materials. *Proceedings of SPIE* **2011**, *8110*, 81100I.

(18) Vandalon, V.; Verheijen, M. A.; Kessels, W. M. M.; Bol, A. A. Atomic Layer Deposition of Al-Doped MoS_2: Synthesizing a P-type 2D Semiconductor with Tunable Carrier Density. *ACS Appl. Nano Mater.* **2020**, *3*, 10200–10208.

(19) Shirazi, M.; Kessels, W. M. M.; Bol, A. A. Initial Stage of Atomic Layer Deposition of 2D-MoS_2 on a SiO_2 Surface: A DFT Study. *Phys. Chem. Chem. Phys.* **2018**, *20*, 16861–16875.

(20) Mackus, A. J. M.; Heil, S. B. S.; Langereis, E.; Knoops, H. C. M.; van de Sanden, M. C. M.; Kessels, W. M. M. Optical Emission Spectroscopy as a Tool for Studying, Optimizing, and Monitoring Plasma-Assisted Atomic Layer Deposition Processes. *J. Vac. Sci. Technol. A.* **2010**, *28*, 77–87.

(21) Sansonetti, J. E.; Martin, W. C. Handbook of Basic Atomic Spectroscopic Data, NIST Standard Reference Database 108 (https://www.nist.gov/pml/handbook-basic-atomic-spectroscopic-data, accessed October, 2021).

(22) Erdevdy, N. M.; Shpenik, O. B.; Markush, P. P. Electron-Impact Excitation of Gas-Phase Sulfur. *J. Appl. Spectrosc.* **2015**, *82*, 19–24.

(23) Jarosz, J.; Mermet, J. M. A Spectroscopic Study of a High-Frequency Inductively-Coupled Ar-H_2S Plasma. *J. Quant. Spectrosc. Radiat. Transf.* **1977**, *17*, 237–246.

(24) Nelson, A. J.; Frigo, S. P.; Rosenberg, R. Surface Type Conversion of $CuInSe_2$ with H_2S Plasma Exposure: A Photoemission Investigation. *J. Vac. Sci. Technol. A* **1995**, *13*, 1990–1993.

(25) Dixon, R. N.; Duxbury, G.; Horani, M.; Rostas, J. The H_2S^+ Radical Ion. A Comparison of Photoelectron and Optical Spectroscopy. *Mol. Phys.* **1971**, *22*, 977–992.

(26) Toyoda, M.; Ogawa, T.; Ishibashi, N. Emission Spectra of Carbon Disulfide, Hydrogen Sulfide, and Thiols by Controlled Electron Impact. *Bul. Chem. Soc. Japan* **1974**, *47*, 95–98.

(27) Ma, H.; Chen, P.; Ruan, R. H_2S and NH_3 Removal by Silent Discharge Plasma and Ozone Combo-System. *Plasma Chem. Plasma Process.* **2001**, *21*, 611–624.

(28) Brotton, S. J.; McConkey, J. W. Dissociative Excitation and Fragmentation of S_8 by Electron Impact. *J. Chem. Phys.* **2011**, *134*, 204301.

(29) Kuhs, J.; Dobbalaere, T.; Hens, Z.; Detavernier, C. Plasma Enhanced Atomic Layer Deposition of Zinc Sulfide Thin Films. *J. Vac. Sci. Technol. A* **2017**, *35*, 01B111.

(30) Kuhs, J.; Hens, Z.; Detavernier, C. Plasma Enhanced Atomic Layer Deposition of Aluminum Sulfide Thin Films. *J. Vac. Sci. Technol. A* **2018**, *36*, 01A113.

(31) Kuhs, J.; Hens, Z.; Detavernier, C. Plasma Enhanced Atomic Layer Deposition of Gallium Sulfide Thin Films. *J. Vac. Sci. Technol. A* **2019**, *37*, 020915.

(32) Delabie, A.; Caymax, M.; Groven, B.; Heyne, M.; Haesevoets, K.; Meersschaut, J.; Nuytten, T.; Bender, H.; Conard, T.; Verdonck, P.; Van Elshocht, S.; De Gendt, S.; Heyns, M.; Barla, K.; Radu, I.; Thean, A. Low Temperature Deposition of 2D WS_2 Layers from WF_6 and H_2S Precursors: Impact of Reducing Agents. *Chem. Commun.* **2015**, *51*, 15692–15695.

(33) AtomicLimits ALD Database, https://www.atomiclimits.com/alddatabase/ (accessed November, 2021).
